# The bacterial iron sensor IdeR recognizes its DNA targets by indirect readout

**DOI:** 10.1093/nar/gkab711

**Published:** 2021-08-20

**Authors:** Francisco Javier Marcos-Torres, Dirk Maurer, Linda Juniar, Julia J Griese

**Affiliations:** Department of Cell and Molecular Biology, Uppsala University, SE-751 24 Uppsala, Sweden; Department of Cell and Molecular Biology, Uppsala University, SE-751 24 Uppsala, Sweden; Department of Cell and Molecular Biology, Uppsala University, SE-751 24 Uppsala, Sweden; Department of Cell and Molecular Biology, Uppsala University, SE-751 24 Uppsala, Sweden

## Abstract

The iron-dependent regulator IdeR is the main transcriptional regulator controlling iron homeostasis genes in Actinobacteria, including species from the *Corynebacterium*, *Mycobacterium* and *Streptomyces* genera, as well as the erythromycin-producing bacterium *Saccharopolyspora erythraea*. Despite being a well-studied transcription factor since the identification of the Diphtheria toxin repressor DtxR three decades ago, the details of how IdeR proteins recognize their highly conserved 19-bp DNA target remain to be elucidated. IdeR makes few direct contacts with DNA bases in its target sequence, and we show here that these contacts are not required for target recognition. The results of our structural and mutational studies support a model wherein IdeR mainly uses an indirect readout mechanism, identifying its targets via the sequence-dependent DNA backbone structure rather than through specific contacts with the DNA bases. Furthermore, we show that IdeR efficiently recognizes a shorter palindromic sequence corresponding to a half binding site as compared to the full 19-bp target previously reported, expanding the number of potential target genes controlled by IdeR proteins.

## Graphical Abstract

**Graphical Abstract:**
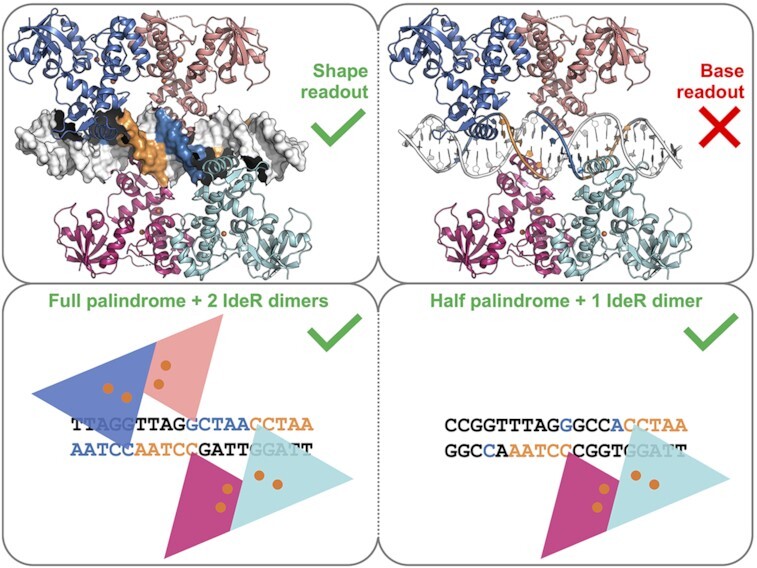
The iron sensor IdeR recognizes its DNA targets, consisting of a 19-bp palindrome binding two IdeR dimers or a 10-bp palindrome binding one dimer, by their sequence-dependent shape rather than through specific contacts with the DNA bases.

## INTRODUCTION

The study of transcriptional regulation in bacteria is critical to our understanding of how microorganisms sense stimuli and how the expression of relevant genes is regulated to adapt to new environmental conditions. *Saccharopolyspora erythraea* is a soil-dwelling actinobacterium best known for producing the macrolide antibiotic erythromycin (1–3). Due to the size of its genome and its multicellular behavior, *S. erythraea* is an excellent bacterial system to study genetic regulation in complex organisms. In the highly variable environment of the soil, bacteria have developed a wide range of sensor systems and signal transduction mechanisms to genetically respond to the stimuli detected by those sensors, for instance, if an essential micro- or macro-nutrient is limiting or if a toxic compound is present.

Bacterial signaling systems have traditionally been classified in four groups, known as the four pillars of signal transduction mechanisms, based on the distribution of their sensory input domains and their DNA-binding effector domains. The simplest signal transduction mechanism is exemplified by the group of one-component systems (OCS), where the sensor and the effector DNA-binding domains are part of the same protein (4–8). With a size of 8.2 Mb, the *S. erythraea* genome encodes 675 DNA-binding proteins predicted to be involved in signal transduction, 652 of which are expected to function as OCS (9). Compared to other less complex bacterial organisms such as *Escherichia coli*, with approximately 250 DNA-binding proteins classified as OCS (9), *S. erythraea* displays a rich assortment of regulatory proteins. Ideally, to avoid cross-talk between all the transcriptional regulators, every DNA-binding protein should specifically recognize a particular target sequence, but in reality, most of these transcriptional regulators recognize different target DNA sequences that resemble a consensus sequence without perfectly matching it. In a bacterium which possesses so many predicted transcriptional regulators, it is of great interest to understand where the boundaries of this pattern recognition flexibility lie and how it can be manipulated to improve or redirect gene regulation. *S. erythraea* in particular is of great biotechnological interest because it produces erythromycin, and much effort has been made to improve production of this secondary metabolite, yet the complex regulation of this process is not completely understood despite decades of research (10–15).

One of the most interesting regulatory processes in bacteria is the one controlling iron homeostasis. As life evolved on Earth, iron became an essential element to almost all organisms because in the primitive oxygen-free environment this transition metal was abundant and soluble in its ferrous (Fe^2+^) form. Iron was incorporated into a variety of enzyme cofactors, since it can be used to transfer electrons, act as a Lewis acid or catalyze redox reactions. As the atmosphere became oxygenated, two major inconveniences arose to which iron-dependent organisms had to adapt. First, iron bioavailability was drastically reduced in all oxygenic environments, as its oxidized ferric (Fe^3+^) form is poorly soluble. Second, iron became highly toxic due to the generation of reactive oxygen species (ROS) through Fenton reactions. Adaptation to these new conditions led to the development of iron uptake and storage mechanisms, including the production of siderophores to complex ferric ions, and to a tight regulation of such mechanisms to avoid the toxicity derived from an excess of iron (16–18). In Gram-negative and Gram-positive bacteria with low GC content, this regulation is usually accomplished by the ferric uptake regulator Fur, whereas in Gram-positive bacteria with high GC content, as well as in archaea, iron homeostasis is frequently controlled by its functional homologue IdeR (iron-dependent regulator) (19,20).

IdeR is an OCS from the DtxR (Diphtheria toxin repressor) family of transcriptional regulators which typically consist of three domains: an N-terminal DNA-binding winged helix-turn-helix (wHTH) motif, followed by a dimerization interface that contains most of the metal-binding residues, and a C-terminal SH3-like domain (21–23). The function of this latter domain remains unclear (discussed further below). It bears structural resemblance, but virtually no sequence similarity to the eukaryotic SH3 (Src homology 3) domains which mediate protein-protein interactions and are commonly found in signaling proteins (24,25).

The main function of IdeR is to repress the expression of iron uptake genes when the intracellular iron levels are sufficient. When intracellular iron levels are low, metal-free IdeR remains inactive, and all iron uptake genes are expressed. When iron levels are sufficient for iron ions to occupy the IdeR metal-binding sites, the regulator is activated and recognizes a highly conserved 19-bp sequence located in the promoter region of its target genes. The binding of IdeR to the promoter generally blocks the transcription of the regulated genes, as is the case for iron uptake genes, thereby preventing the iron concentration from reaching toxic levels. On the other hand, it has also been reported that IdeR can activate the transcription of some genes, such as iron storage genes, in response to high iron concentrations (26–31).

The structural and functional details of the DNA-binding mechanism of this regulator are still not fully understood. By screening the ability of IdeR from *S. erythraea* (*Se*IdeR) to bind to variations of a DNA target, and analyzing the structural details of those interactions, we provide an in-depth description of the specificity of this transcriptional regulator and the thresholds of its tolerance for recognizing a particular DNA pattern, unveiling which regulator residues are involved in this process and which DNA bases provide the specific fingerprint being recognized. We show that IdeR recognizes half binding sites, expanding the already vast repertoire of putative binding sites for this type of regulator. We also provide evidence that IdeR uses an indirect readout mechanism to recognize its DNA targets, identifying them by their sequence-dependent backbone structure rather than through contacts with the DNA bases themselves. The similarities of the wHTH motif with other IdeR proteins implies that other members of this family of bacterial transcriptional regulators also use an indirect readout mechanism to find their targets.

## MATERIALS AND METHODS

### Identification of IdeR binding sites

To identify the putative targets of IdeR in the genome of *S. erythraea*, a pattern search was performed using the Pattern Locator software developed by CMBL (https://www.cmbl.uga.edu/software/patloc.html) (32), searching for the full 19-bp consensus sequence with a 6 mismatch allowance, as well as the half binding site with only one mismatch. The resulting hits were manually curated by discarding all non-intergenic sequences and sequences located further than 500 bp from the closest annotated starting codon. A total of 37 sequences were selected from the resulting list as likely IdeR targets, either by sequence conservation, redundancy in their gene cluster, or by predicted product.

### Cloning

The full-length *S. erythraea* IdeR (accession number WP_009947362.1) coding sequence was PCR-amplified from genomic DNA (DSM number 40517) and inserted into a modified version of pET-28a(+) (Novagen), which encodes the recognition sequence for Tobacco Etch Virus (TEV) protease instead of thrombin, using the NdeI and HindIII restriction sites (see Supplementary Table S1 for primer sequences). The resulting construct was verified by DNA sequencing. It encodes full-length IdeR with a TEV-cleavable N-terminal hexahistidine tag and no C-terminal tag, so that after TEV cleavage, full-length IdeR including the N-terminal Met residue remains with two additional N-terminal amino acids (Gly-His). Point mutations (Q43A, P39G) were introduced into this construct by PCR-based site-directed mutagenesis using the QuikChange™ method and verified by DNA sequencing (see Supplementary Table S1 for primer sequences).

### Protein production and purification

*E. coli* BL21(DE3) (Novagen) cells transformed with the plasmid encoding IdeR^WT^ or one of its engineered variants IdeR^Q43A^ or IdeR^P39G^ were grown in terrific broth (TB) medium supplemented with 50 μg/ml kanamycin at 37°C to an OD_600_ of ∼0.5. Expression was then induced by adding 0.1 mM isopropyl‐β‐d‐thiogalactopyranoside (IPTG) and the cultures were harvested after over-night incubation at 20°C. The harvested cells were resuspended in IdeR lysis buffer (25 mM 2-(*N*-morpholino)ethanesulfonic acid [MES] pH 6.0, 450 mM NaCl, 10% (v/v) glycerol) with the addition of 5 mM MgSO_4_, 1 mM phenylmethylsulfonyl fluoride (PMSF), DNase and lysozyme, and lysed with a cell disruptor (Constant Systems). The lysate was cleared by centrifugation and the supernatant was applied to a gravity flow column containing Ni^2+^-charged immobilized metal ion affinity chromatography (IMAC) resin (Ni Sepharose 6 Fast Flow, Cytiva). The column was washed with at least 10 column volumes (CV) IdeR wash buffer (25 mM MES pH 6.0, 450 mM NaCl, 60 mM imidazole, 10% (v/v) glycerol) and the protein eluted with 5 CV IdeR elution buffer (25 mM MES pH 6.0, 450 mM NaCl, 500 mM imidazole, 10% (v/v) glycerol). The eluted protein sample was then concentrated to an appropriate volume and exchanged into IdeR lysis buffer using PD10 columns (Cytiva). The His-tag of the recombinant IdeR was cleaved over-night at room temperature by adding 0.5 mM EDTA, 10 mM β-mercaptoethanol and TEV protease at a ratio of 1 μM TEV per 100 μM IdeR monomer. The digested protein sample was diluted with IdeR lysis buffer to decrease the β-mercaptoethanol concentration below 5 mM, and imidazole was added to a final concentration of 60 mM. The sample was then again applied to a Ni^2+^-charged IMAC gravity flow column and the flow-through, containing the tag-free IdeR, collected. The column was washed with 5 CV of IdeR wash buffer and the flow-through and wash fractions were combined and concentrated to a protein concentration of ∼0.8 mM (20 mg/ml). Protein concentration was determined using a calculated extinction coefficient at 280 nm of 15.47 mM^–1^ cm^–1^ for the IdeR monomer (33). The protein was then aliquoted, flash-frozen in liquid nitrogen and stored at -80°C until further use.

His-tagged TEV protease (34) was produced and purified similarly as IdeR, with the following differences. *E. coli* BL21(DE3) (Novagen) cells transformed with the plasmid encoding TEV (34) were grown in TB medium supplemented with 50 μg/ml ampicillin, and expression was induced with 1 mM IPTG. TEV lysis buffer contained 50 mM Tris–HCl pH 8.0, 200 mM NaCl and 10% (v/v) glycerol. The cleared lysate was incubated with Ni^2+^-charged IMAC resin for 1 h at 4°C before the slurry was transferred into gravity flow columns and washed with at least 10 CV TEV wash buffer (50 mM Tris–HCl pH 8.0, 200 mM NaCl, 10% (v/v) glycerol, 60 mM imidazole). TEV protease was eluted with 5 CV TEV elution buffer (50 mM Tris–HCl pH 8.0, 200 mM NaCl, 10% (v/v) glycerol, 300 mM imidazole), concentrated to an appropriate volume, and exchanged into TEV lysis buffer using PD10 columns (Cytiva). The purified protease was then again concentrated to ∼200 μM, following which EDTA was added to a final concentration of 2 mM, dithiothreitol to 5 mM, and glycerol to a final concentration of 50% (v/v), so that the final concentration of TEV protease was ∼100 μM. TEV protease concentration was determined using an extinction coefficient at 280 nm of 36.13 mM^–1^ cm^–1^ (34). TEV protease was aliquoted, flash-frozen in liquid nitrogen and stored at -80°C until further use.

### Total-reflection X-ray fluorescence (TXRF) analysis of protein metal contents

Metal contents of IdeR protein preparations were quantified using total-reflection X-ray fluorescence (TXRF) analysis on a Bruker S2 PicoFox instrument (35). A gallium standard (Sigma) was added to the samples (v/v, 1:1) prior to the measurements. Technical duplicates were prepared of each sample. TXRF spectra were analyzed using the routines provided with the spectrometer. The IdeR batches as purified contained ∼13% Ni and ∼17% Fe and negligible amounts of other transition metal ions (∼1% each Zn and Mn and 0.2% Cu).

### Preparation of double-stranded DNA

Forward and reverse DNA oligonucleotides containing the different target sequences designed for DNA-binding analysis and crystallization studies were obtained from Eurofins or Thermo Fisher Scientific with or without a 5′ Cy5 or FAM fluorescent label (see Supplementary Table S1). To prepare double-stranded DNA, each forward and reverse oligonucleotide sample pair was resuspended in DNA buffer (40 mM Tris–HCl pH 7.4, 100 mM NaCl, 10 mM MgSO_4_) and mixed to a final concentration of 375 μM (for co-crystallization with IdeR) or 10 μM (for EMSA) each. All mixtures were then heated at 95°C for 10 min and slowly cooled down to 4°C in a thermal cycler.

### Electrophoretic mobility shift assays (EMSAs)

**Figure 1.**
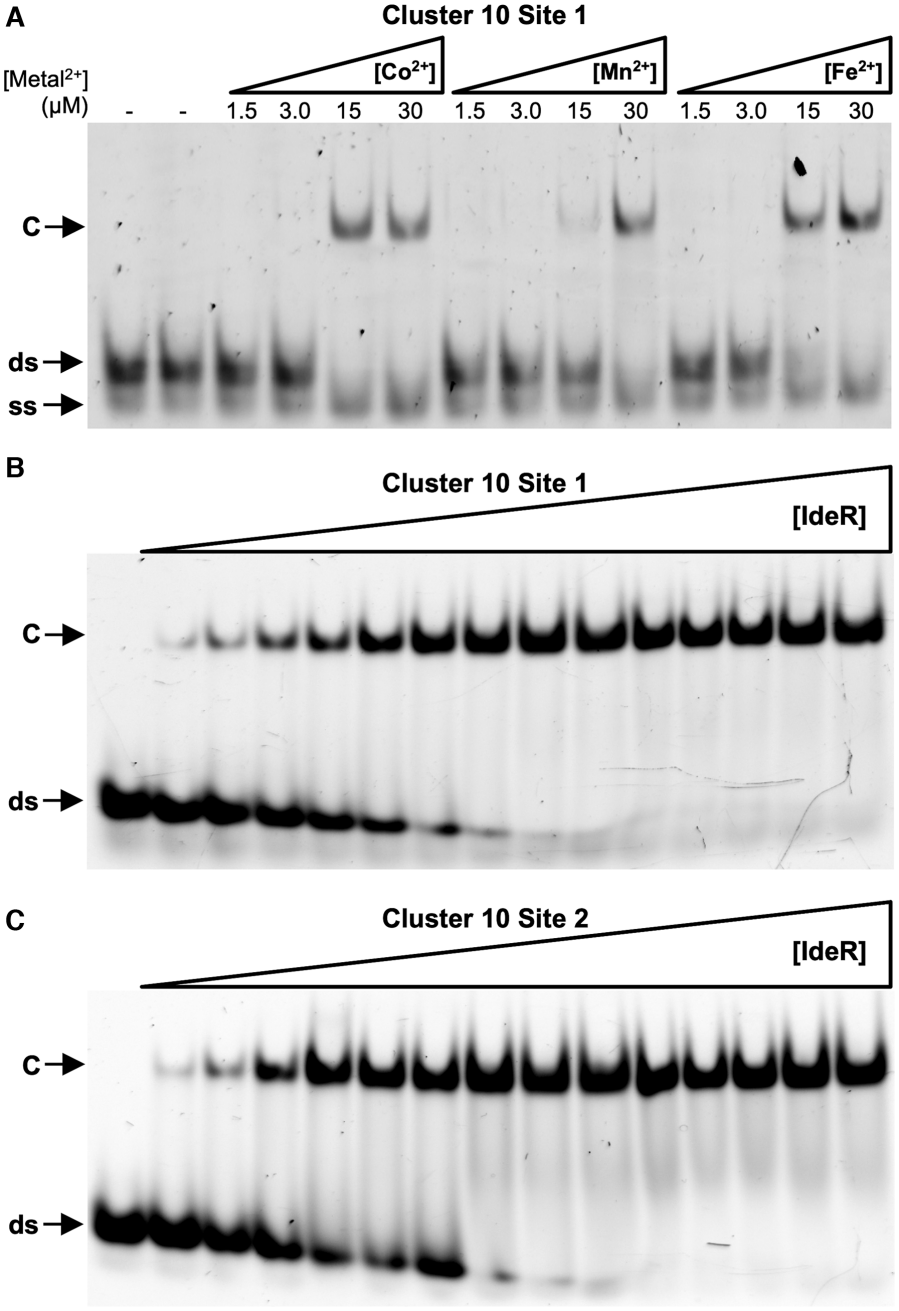
EMSA analysis of IdeR binding to native *S. erythraea* DNA-binding sites. (**A**) Binding of IdeR at 750 nM (dimer concentration) to site 1 of cluster 10 in the presence of increasing concentrations of Co^2+^, Mn^2+^, or Fe^2+^. (**B**, **C**) Binding of IdeR, in increasing concentrations (15 nM–2.25 μM dimer), to (**B**) site 1 of cluster 10 or (**C**) site 2 of cluster 10 in the presence of 30 μM Co^2+^. IdeR was added to 30 nM fluorescence-labeled double-stranded DNA probe in the presence of competitor DNA. Protein–DNA complexes were resolved on a 4% Tris-acetate polyacrylamide gel. The left-most lane is a control reaction without protein. ds, unbound double-stranded DNA probe; ss, non-hybridized single-stranded DNA; C, protein–dsDNA complex.

EMSAs were performed after mixing the fluorescently-labelled double-stranded DNA samples at 30 nM with IdeR protein samples and MnCl_2_, Fe(NH_4_)_2_(SO_4_)_2_ or CoCl_2_ at different concentrations (see legends to Figures 1, 3, 5 and 6 and Supplementary Figure S5 for the protein and metal ion concentrations used in each assay). Fe(NH_4_)_2_(SO_4_)_2_ was freshly dissolved with an excess of ascorbic acid to ensure that the iron remained ferrous prior to addition to the samples. All mixtures were prepared in TAKA buffer (15 mM Tris-acetate pH 7.3, 4 mM potassium acetate), containing glycerol at a final concentration of 10% and using poly(dI-dC) (poly(deoxyinosinic-deoxycytidylic) acid; Thermo Fisher Scientific) as competitor DNA at a final concentration of 17 ng/μl, and incubated at room temperature for 20 min. Each EMSA was run at 4°C in a 4% native polyacrylamide gel for 30–40 min at 20 mA. Fluorescence of the unbound DNA and the DNA bound to IdeR was then observed in a BioRad ChemiDoc MP Imager at the proper wavelength for each of the labels. EMSAs were performed at least twice and using different protein concentrations to ensure reproducibility. For the Consensus_Full sequence, both 5′ Cy5 and 5′ FAM labels were tested to exclude effects of the label on IdeR binding (see Supplementary Table S1). For the estimation of the apparent dissociation constants (*K*_D_), the band intensities in the EMSA gel images were estimated using ImageJ (https://imagej.nih.gov/ij/) (36), the relative amount of bound and unbound DNA was calculated from the band intensities, and the resulting data was fit to the Hill–Langmuir equation using MATLAB.

### Crystallization and data collection

Crystallization conditions were screened using commercial kits (Molecular Dimensions) in sitting-drop vapor diffusion setups at 20°C using a Mosquito^®^ Crystal liquid handling robot (SPT Labtech), followed by optimization of the identified conditions.

To obtain the structure of IdeR^WT^ complexed with cobalt, a 12 mg/ml sample of IdeR^WT^ was mixed with 1 mM CoCl_2_ and 0.3 mg/ml trypsin and incubated at room temperature for 30 min. The digested sample was flash-frozen in liquid nitrogen and stored at –80°C until the next day. The protein was then crystallized in a sitting-drop vapor diffusion experiment at 20°C by mixing 110 nl of the digested sample with 90 nl 25% (w/v) PEG 1500 using the Mosquito^®^ Crystal robot. Crystals were flash-cooled in liquid nitrogen without the addition of cryoprotectant. A dataset was collected at 100 K at beamline I04 of the Diamond Light Source (Didcot, UK) (Table 1).

**Table 1.** Crystallographic data and refinement statistics

Structure	Co^2+^-IdeR^WT^	Co^2+^-IdeR^WT^ + consensus DNA	Fe^2+^-IdeR^WT^ + consensus DNA	Co^2+^-IdeR^WT^ + C10S1 DNA	Co^2+^-IdeR^P39G^ + consensus DNA	Co^2+^-IdeR^Q43A^ + consensus DNA
PDB ID	7B1V	7B1Y	7B20	7B23	7B24	7B25
**Data collection and processing**						
Wavelength	0.980	0.976	0.976	0.918	0.976	0.976
Resolution (Å)	61.10–2.04 (2.22–2.04)	78.94–2.12 (2.32–2.12)	54.29–2.18 (2.44–2.18)	94.63–2.15 (2.41–2.15)	54.93–2.05 (2.23–2.05)	93.16–2.34 (2.63–2.34)
Resolution limits along axes (a*, b*, c*)	2.58, 2.04, 2.21	2.12, 2.18, 3.06	2.41, 2.18, 2.95	2.15, 2.48, 2.72	2.05, 2.13, 2.65	2.32, 2.81, 3.34
Space group	*P*1	*C*2	*C*2	*C*2	*C*2	*C*2
Cell dimensions						
* a, b, c* (Å)	43.88, 45.56, 66.28	195.04, 112.90, 88.51	194.12, 112.66, 88.55	194.25, 113.13, 89.29	196.40, 113.84, 89.80	192.66, 110.86, 86.75
α, β, γ (°)	109.12, 93.20, 113.98	90.00, 117.07, 90.00	90.00, 117.28, 90.00	90.00, 117.25, 90.00	90.00, 117.25, 90.00	90.00, 116.85, 90.00
No. unique reflections	17 216 (862)	60 545 (3027)	54 157 (2708)	59 657 (2984)	76 685 (3835)	36 928 (1847)
Multiplicity	3.3 (2.4)	6.9 (6.7)	6.9 (6.7)	6.7 (6.7)	7.0 (6.9)	6.8 (6.5)
*R*_merge_	0.058 (0.579)	0.092 (1.203)	0.093 (1.136)	0.083 (0.984)	0.082 (1.305)	0.130 (1.218)
*R*_meas_	0.069 (0.748)	0.099 (1.304)	0.100 (1.229)	0.090 (1.066)	0.088 (1.411)	0.141 (1.323)
*R*_pim_	0.037 (0.465)	0.038 (0.497)	0.038 (0.463)	0.035 (0.406)	0.033 (0.532)	0.054 (0.511)
*I/σ(I)*	10.3 (1.5)	11.2 (1.7)	11.4 (1.7)	10.9 (1.6)	12.0 (1.7)	9.1 (1.8)
Completeness (%)						
spherical	62.7 (13.9)	62.8 (13.7)	61.4 (10.8)	63.8 (11.1)	70.2 (16.3)	53.9 (9.1)
ellipsoidal	83.5 (54.2)	92.5 (59.0)	91.1 (59.7)	92.6 (71.0)	93.1 (61.6)	89.5 (61.1)
CC_1/2_	0.997 (0.658)	0.997 (0.581)	0.998 (0.516)	0.998 (0.604)	0.998 (0.573)	0.997 (0.599)
**Refinement**						
Resolution (Å)	61.10–2.04	78.94–2.12	54.29–2.18	94.63–2.15	54.93–2.05	93.16–2.34
No. reflections	16 378	57 538	51 311	56 713	72 891	35 059
*R*_work_/*R*_free_ (%)^a^	23.2/26.2	23.0/25.3	20.9/24.6	22.6/24.8	22.6/25.9	26.7/28.3
No. non-H atoms	3491	8335	8350	8203	8310	8256
Protein	3420	7002	7048	6956	6921	7016
DNA	–	1189	1189	1189	1189	1189
Metal ions	4	8	8	8	8	8
Water	67	136	105	50	192	43
*B*-factors						
Protein	38.4	51.9	60.5	60.8	51.1	65.1
DNA	–	60.9	68.3	72.1	74.4	78.4
Metal ions	21.3	38.4	50.2	44.6	39.6	46.8
Water	30.7	41.1	47.3	45.5	47.9	39.2
Overall	35.8	50.2	57.9	58.1	51.3	62.7
R.m.s. deviations						
Bond lengths (Å)	0.0017	0.0020	0.0021	0.0022	0.0019	0.0032
Bond angles (°)	1.149	1.100	1.096	1.117	1.099	1.146
Ramachandran favored/outliers (%)^b^	97.4/0.0	97.4/0.0	97.7/0.0	97.1/0.0	98.5/0.0	96.9/0.0
Rama *Z*-score^b^	–1.88 ± 0.34	–1.44 ± 0.24	–1.59 ± 0.23	–1.80 ± 0.24	–1.22 ± 0.24	–2.09 ± 0.22
Rotamers favored/poor (%)^b^	98.7/0.5	97.1/0.0	98.1/0.0	97.6/0.0	98.4/0.0	97.2/0.0
Clashscore^b^	0.58	0.39	0.45	0.92	0.59	1.37
MolProbity score^b^	0.81	0.76	0.73	0.93	0.70	1.06

Values in parentheses are for the highest resolution shell. Friedel pairs were merged. ^a^*R*_free_ is calculated from a randomly selected subset of ∼5% of reflections exluded from refinement. ^b^ Geometry statistics were calculated with MolProbity (45).

To obtain structures of protein–DNA complexes, a 10 mg/ml protein solution, containing 1 mM CoCl_2_ or Fe(NH_4_)_2_(SO_4_)_2_ and 150 μM double-stranded DNA (see Supplementary Table S1), was incubated at room temperature for 10 min. For Co^2+^-activated and Fe^2+^-activated IdeR^WT^ complexed with consensus DNA, Co^2+^-activated IdeR^WT^ with C10S1 DNA, as well as the Co^2+^-activated P39G variant in complex with consensus DNA, this solution was then mixed with crystallization solution containing 30% (w/v) PEG 2000 monomethyl ether, 200 mM ammonium sulfate and 100 mM sodium acetate at pH 4.6 in a sitting-drop vapor diffusion experiment at 20°C using the Mosquito^®^ Crystal robot. The total drop volume was 200 nl and the protein volume was 67 nl, 100 nl or 133 nl. Crystals of the Co^2+^-activated IdeR^Q43A^-consensus DNA complex were obtained in a hanging-drop vapor diffusion experiment at 20°C by manually mixing 1.2 μl protein solution with 0.8 μl crystallization solution and 0.4 μl seed stock consisting of microcrystals of the same protein–DNA complex. The crystallization solution contained 29% (w/v) PEG 3350, 280 mM ammonium sulfate and 100 mM MES at pH 6.5. Crystals were flash-cooled in liquid nitrogen without the addition of cryoprotectant. All datasets of IdeR-DNA complexes were collected at 100 K at the BioMAX beamline of the MaxIV laboratory (Lund, Sweden) (Table 1).

### Structure determination, model building and refinement

All datasets were processed with the autoPROC toolbox (37) including the STARANISO server (http://staraniso.globalphasing.org/cgi-bin/staraniso.cgi), as well as XDS (38), POINTLESS (39), AIMLESS (40) and other CCP4 programs (41). Since diffraction was significantly anisotropic in all cases, elliptical diffraction cut-offs were chosen using STARANISO based on the criterion that the local *I*/σ(*I*) ≥1.20. Co^2+^-activated IdeR crystallized in space group P1 with one IdeR dimer in the asymmetric unit. The structure was solved by molecular replacement using PHASER (42) and chain A of the structure of *Mycobacterium tuberculosis* IdeR (PDB ID 1U8R) (22) as search model. All DNA complexes were in space group C2 with two IdeR dimers and one DNA duplex in the asymmetric unit, with an NCS rotation axis along the DNA double helix axis leading to I222 pseudosymmetry. The DNA complex structures were solved by molecular replacement using the *Se*IdeR monomer structure as a search model, and the DNA chains were manually built in Coot (43). Refinement was carried out with REFMAC5 (44) and iterated with rebuilding in Coot. Refinement included bulk solvent corrections, individual atomic coordinate and isotropic *B* factor refinement. For the structure of the Co^2+^-IdeR^Q43A^-consensus DNA complex, external restraints were applied to protein and DNA chains based on the final model of the Fe^2+^-IdeR^WT^-consensus DNA complex in the final step of refinement. This was necessary to maintain both acceptable model geometry and fit to the data due to the significantly lower resolution of this dataset along the b* and c* axes compared to the other datasets (see Table 1). Metal–ligand bond lengths were not restrained and riding hydrogens were used during refinement. Solvent molecules were added with the ‘find waters’ function in Coot and manually curated. Structures were validated using MolProbity (http://molprobity.biochem.duke.edu/) (45). Data and refinement statistics are given in Table 1. The DNA conformation in the crystal structures was analyzed with the Curves + web server (46,47), and protein-DNA interactions were analyzed with DNAproDB (48). All figures were prepared with PyMOL (version 2.4.1; Schrödinger, LLC).

### Prediction of DNA features

The DNAshape and DNAphi web servers were used to predict four structural features and the electrostatic potential in the minor groove, respectively, of the DNA sequences used for EMSAs (49,50).

## RESULTS

### IdeR likely controls the expression of at least 23 gene clusters in *S. erythraea*

The DNA-binding wHTH motif of *Se*IdeR shares an amino acid identity of around 92%, 96% and 100% with those of IdeR from *Mycobacterium tuberculosis* and *Streptomyces avermitilis*, and DtxR from *Corynebacterium diphtheriae*, respectively. Considering that these proteins recognize a similar 19-bp DNA target in their respective hosts (22,30,51), we used the 19-bp consensus sequence to screen the genome of *S. erythraea* for putative IdeR targets. This search provided >70 sequences which were manually curated to obtain 37 reliable putative binding sites that could be matched to 23 gene clusters (Supplementary Table S2).

Most of the identified gene clusters are involved in the uptake and storage of iron, with a high presence of genes coding for siderophore production or transport, iron ABC transport systems and bacterioferritins, as well as an EfeO-like ferric iron uptake transporter. Additionally, IdeR appears to regulate several gene clusters encoding proteins that use iron as a cofactor. Among these we find ferredoxins, an L-lactate dehydrogenase, and the Nuo NADH dehydrogenase complex of the respiratory chain (Supplementary Table S2). Notably, IdeR does not seem to be subject to autoregulation at the level of transcription, as we were unable to identify an IdeR binding site in the promoter region of the *ideR* gene.

To confirm some of the putative binding sites listed in Supplementary Table S2, we analyzed the binding of IdeR to two of the binding sites found in cluster 10. This cluster is predicted to be involved in the production and transport of a siderophore to capture environmental iron. The first two putative IdeR binding sites in the cluster (C10S1 and C10S2) can be found in the intergenic region between genes SACE_2689 and SACE_2690. The orientation of both genes suggests the presence of a divergent promoter in this intergenic region, with C10S1 being closer to the start codon of gene SACE_2689, and C10S2 to the start codon of SACE_2690. To assess IdeR binding to its target sequences in different metalation conditions, we performed an electrophoretic mobility shift assay (EMSA) using 30 nM of fluorescein-labelled C10S1 DNA in the presence of 25 times excess of IdeR and different metal concentrations. As can be seen in Figure 1A, IdeR binds to C10S1 in the presence of Co^2+^, Mn^2+^, and Fe^2+^ starting at concentrations of 15 μM. However, the activation by Mn^2+^ does not appear to be as efficient as by Co^2+^ or Fe^2+^, which activate IdeR at similar concentrations, as previously reported for *M. tuberculosis* IdeR and *C. diphtheriae* DtxR (52–56). Due to the similarities between both metals (57), and the constraints of working with Fe^2+^, we used Co^2+^ to activate IdeR in all of the subsequent DNA-binding experiments.

The affinity of Co^2+^-activated IdeR for the C10S1 and C10S2 DNA targets was assessed by EMSA using increasing concentrations of IdeR. IdeR binds specifically to both DNA sequences, with similar apparent dissociation constants (*K*_D_), which are estimated to be around 116 nM and 94 nM for the C10S1 and C10S2 targets, respectively (Figure 1B and C, Table 2). These *K*_D_ values are in good agreement with those previously reported for IdeR from *M. tuberculosis* (54,58).

**Table 2.** DNA sequences used for EMSA analysis and estimated apparent dissociation constant (*K*_D_) for each binding reaction with IdeR^WT^

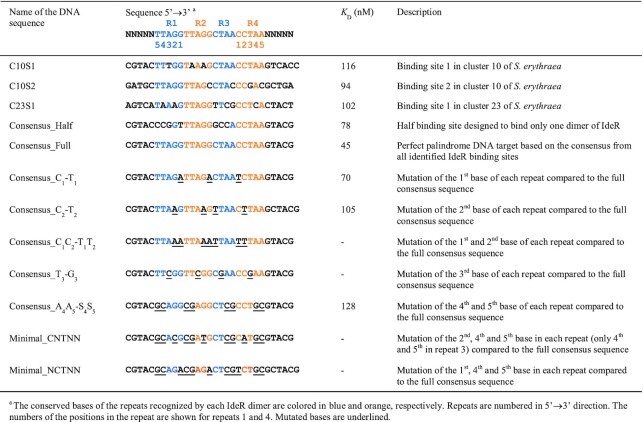

### Two IdeR dimers bind to the palindromic recognition sequence

**Figure 2.**
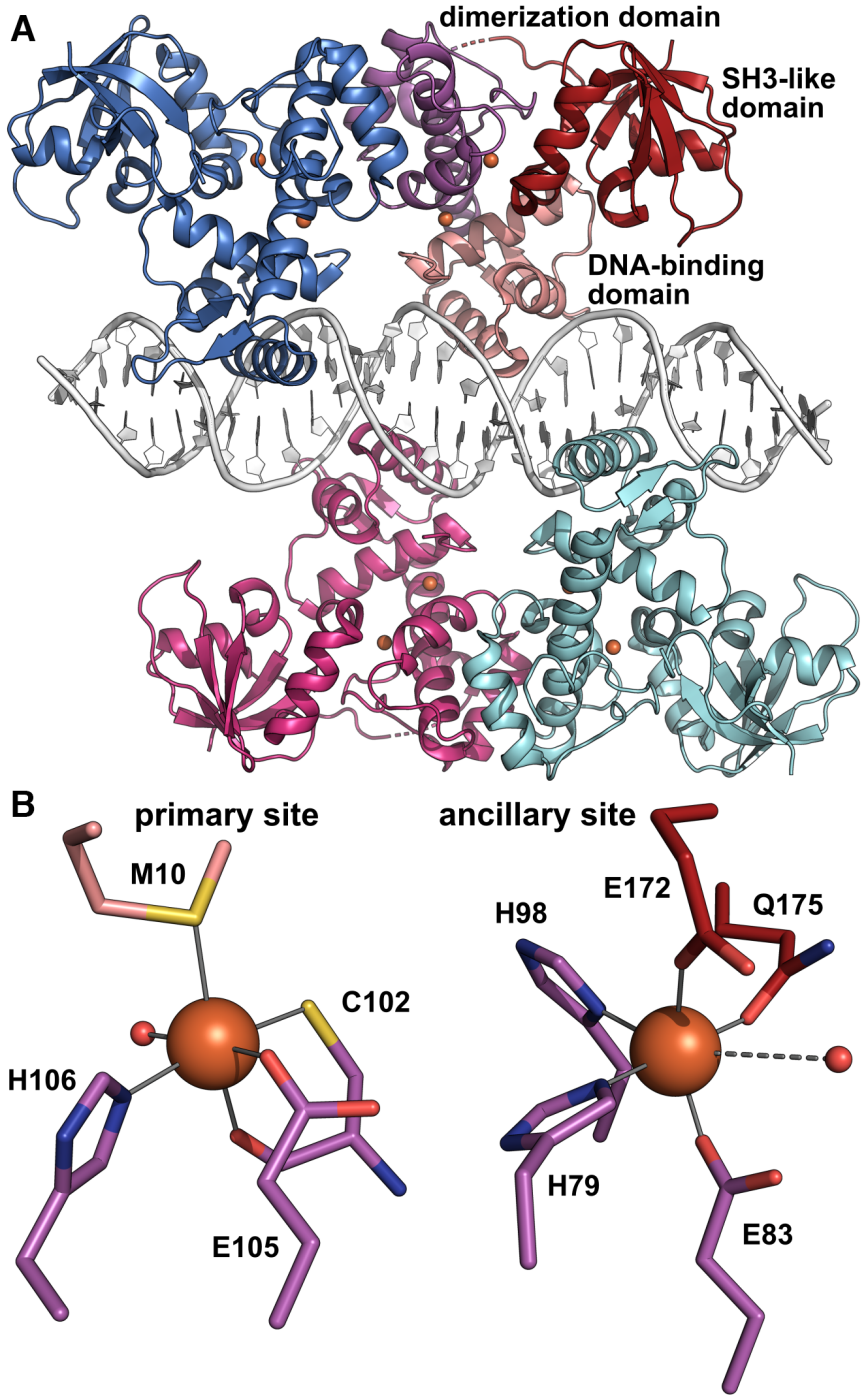
Crystal structure of Fe^2+^-activated IdeR in complex with the consensus DNA-binding sequence. (**A**) Overall structure of the complex, with IdeR subunit B colored by domain and the other IdeR subunits colored by subunit. The Fe^2+^ ions are shown as orange spheres. Two IdeR dimers bind to opposite faces of the DNA double helix. (**B**) The metal-binding sites in IdeR subunit B, depicted using the same coloring scheme as in panel A, with water ligands shown as small red spheres. Metal-ligand bonds are indicated by grey lines, the dashed line between the ancillary site metal ion and water ligand indicating a long, weak bond.

We determined the crystal structure of Co^2+^-activated IdeR at 2.0 Å resolution (Table 1, Supplementary Figure S1A, see also Figure 2A). The protein forms a dimer. Each subunit consists of the three domains typical of an IdeR protein, an N-terminal domain containing the DNA-binding wHTH motif, a dimerization domain, and a C-terminal SH3-like domain. Two metal-binding sites are formed by residues from all three domains, coordinating two Co^2+^ ions in octahedral geometry (Supplementary Figure S2A, see also Figure 2B). The overall structure of *Se*IdeR is highly similar to the structures of IdeR from *M. tuberculosis* (22) and DtxR from *C. diphtheriae* (59) (Supplementary Table S3).

We then co-crystallized IdeR with a 30-bp double-stranded DNA oligomer containing the consensus sequence TTAGGTTAGSCTAACCTAA (S = G or C, i.e. G in the forward strand and C in the reverse strand; see Table 2 and Supplementary Table S1). Crystal structures were obtained for the complexes with the physiological activator Fe^2+^ as well as the mimic Co^2+^ (Table 1). Both crystallized in space group C2, containing four polypeptide chains forming two dimers and two DNA strands forming a distorted B-type double helix in the asymmetric unit (Figure 2A, Supplementary Figures S1B and S3A). Each IdeR subunit binds to one of the four CCTAA repeats of the recognition sequence, one dimer interacting with repeats 1 and 3 and the other with repeats 2 and 4 (see Table 2). Both metal-binding sites of each IdeR subunit are occupied in both the Fe^2+^ and Co^2+^ complexes (Figure 2B, Supplementary Figure S2B and C). The structures of these IdeR dimers are very similar to the DNA-free dimer structure (Supplementary Table S3). DNA binding primarily causes a slight shift of the recognition helices, which is necessary to allow these helices to insert into the major grooves of the DNA (Supplementary Figure S1A). No significant differences between the Fe^2+^-activated and Co^2+^-activated IdeR-DNA complexes can be discerned, neither globally, nor at the metal-binding sites (Supplementary Figures S1B and S2B and C, Supplementary Table S3). Interestingly, in the DNA complex structures we observe a swap of the SH3-like domains of one subunit of each IdeR dimer with a symmetry-related chain (Supplementary Figure S3B).

### IdeR recognizes half binding sites

Both DNA sequences evaluated above (Figure 1) have a conserved IdeR binding site, with only three and two mismatches compared to the perfect palindromic consensus, respectively (Table 2). However, some of the predicted targets collected in Supplementary Table S2, such as C23S1, diverge more from the full consensus. The binding site at C23S1 is predicted to control the expression of the complex I NADH dehydrogenase of the respiratory chain. This sequence has 6 mismatches with the full consensus, and the distribution of those mismatches suggests that it can only be recognized by one IdeR dimer, instead of the typical two dimers (Table 2).

**Figure 3.**
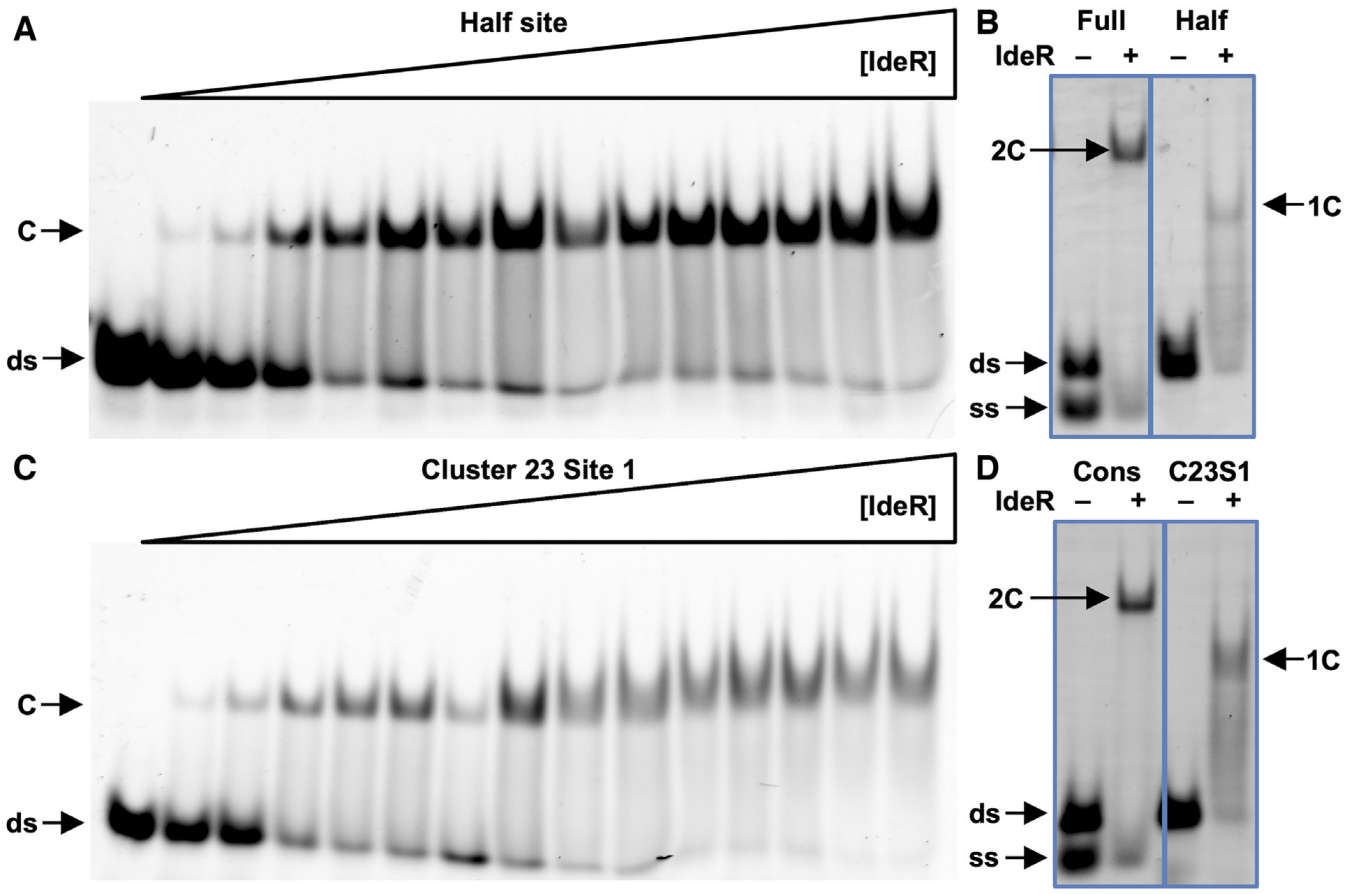
EMSA analysis of the stoichiometrical requirement of IdeR to bind its target DNA. (**A**) Binding of IdeR to a DNA sequence designed to bind only one IdeR dimer. (**B**) Binding of IdeR to the full consensus DNA sequence and the designed half binding site. (**C**) Binding of IdeR to site 1 of cluster 23. (**D**) Binding of IdeR to the full consensus DNA sequence and site 1 of cluster 23. IdeR was added to 30 nM fluorescence-labeled double-stranded DNA probe in (A, C) increasing concentrations (15 nM–2.25 μM dimer) or (B, D) at 1.5 μM in the presence of (A, C) 30 μM or (B, D) 40 μM Co^2+^ and competitor DNA. Protein–DNA complexes were resolved on a 4% Tris-acetate polyacrylamide gel. The left-most lane in panels A and C is a control reaction without protein. The gel images in panels B and D have been edited for easier comparison between both samples. ds, unbound double-stranded DNA probe; ss, non-hybridized single-stranded DNA; C, protein–dsDNA complex; 2C, protein–dsDNA complex containing two IdeR dimers; 1C, protein–dsDNA complex containing one IdeR dimer.

To date, all described IdeR/DtxR complexes with DNA involve two dimers bound to the 19-bp target sequence (22,29,58–60). To test if the binding of only one dimer is possible with only half of the DNA target, we designed a DNA sequence harboring the two CCTAA motifs that should be recognized by one of the dimers while disrupting the sequence that should be bound by the second dimer. As can be seen in Figure 3A, IdeR is able to bind to this half binding site with an estimated apparent *K*_D_ of 78 nM, comparable to the affinity of IdeR for the previously tested C10S1 and C10S2 DNA targets (Table 2). A comparison of the electrophoretic mobility of IdeR complexes with a complete DNA target and with the half binding site clearly shows that only one IdeR dimer is bound to the half site target (Figure 3B).

IdeR also recognizes the C23S1 DNA target with an estimated apparent *K*_D_ of 102 nM (Figure 3C, Table 2). As expected from the sequence analysis, the binding stoichiometry of this complex is of only one dimer per DNA molecule, forming a complex similar to that observed with the half binding site target (Figure 3D). These results indicate that the DNA targets of IdeR do not require to be recognized by two dimers, and expand the number of putative targets beyond what was previously predicted for this family of regulators.

### IdeR forms specific interactions with only three out of five DNA bases in the recognition sequence

**Figure 4.**
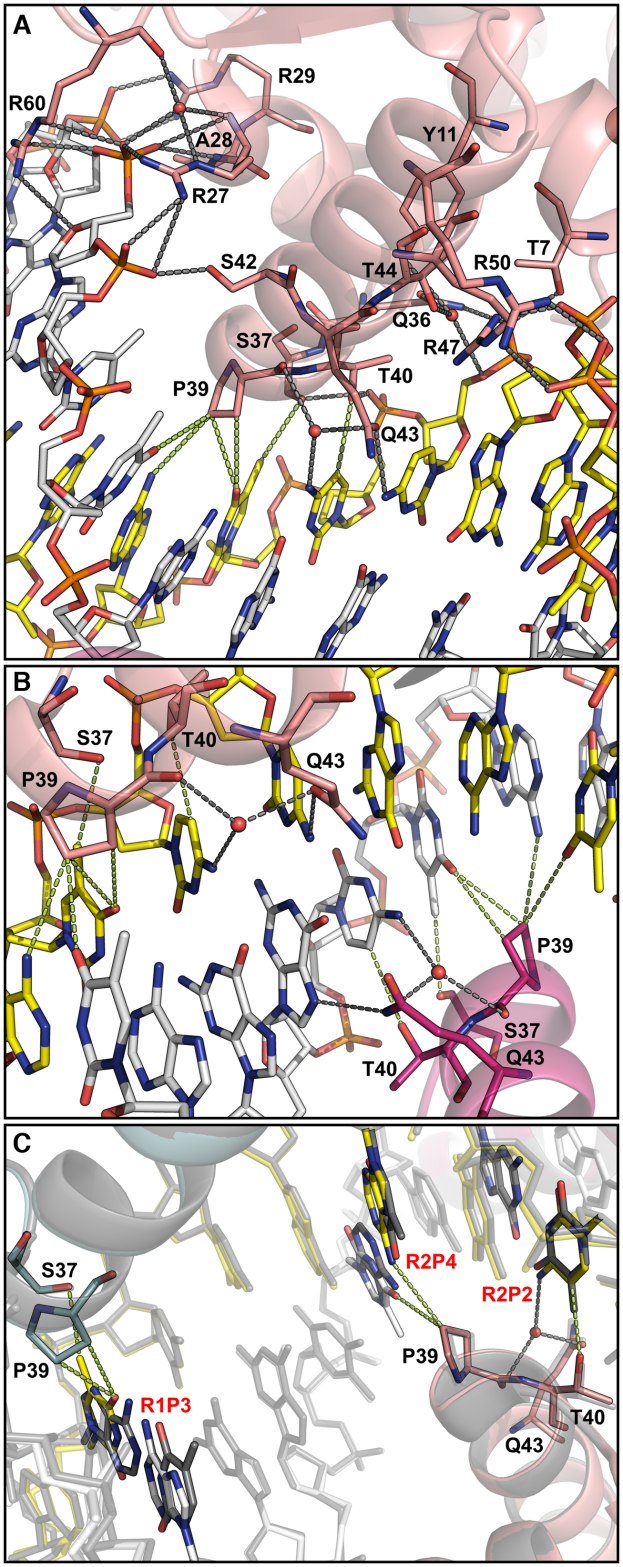
Interactions between IdeR and DNA. (**A**) Interactions between each IdeR subunit and the consensus DNA recognition sequence, illustrated with IdeR subunit B of the Fe^2+^-activated IdeR-consensus DNA complex. In every subunit, the side chains of Thr7, Arg27, Arg29, Gln36, Ser37, Thr40, Ser42, Arg47, Arg50 and Arg60, as well as the peptide bond amide group of Ala28 form hydrogen bonds and salt bridges with the sugar-phosphate backbone of the DNA. Additionally, water-mediated interactions with phosphates are formed by the side chains of Tyr11, Gln36 and Thr44, as well as the backbone amide of Arg27 and the backbone carbonyl group of Arg60. Arg60 in the wing of the wHTH motif is the only residue interacting with the DNA backbone on the minor groove edge. In one subunit of each dimer it interacts with a phosphate group and a ribose (as shown here), while in the other it adopts a different rotamer and only binds to the phosphate. Gln43 forms a hydrogen bond with the first cytosine in the CCTAA repeat of the palindromic recognition sequence as well as a water-mediated hydrogen bond with the second cytosine, which also forms vdW interactions with Thr40. Ser37 and Pro39 form vdW interactions with the thymine in position 3. Pro39 also interacts with the fourth basepair in the repeat. (**B**) Interactions between Fe^2+^-activated IdeR and DNA bases, focused on the central G–C basepair in the consensus DNA-binding sequence, which interacts with two IdeR subunits from different dimers. (**C**) Comparison of the interactions between IdeR and the DNA that are affected by the differences between the consensus sequence and the C10S1 sequence. The Fe^2+^-activated IdeR-consensus DNA complex is shown colored by subunit, while the Co^2+^-activated IdeR-C10S1 DNA complex is shown in transparent grey. For clarity, the DNA strands in both complexes are shown partially transparent, except for the bases that differ between the two DNA sequences, which are also colored by element. Ser37, Pro39 and Gln43 of the IdeR subunits that interact with the differing bases are shown as sticks. Interactions that are affected by the sequence differences are shown only for the consensus sequence. The C10S1 sequence differs from the consensus in the third position of the first repeat (R1P3, A instead of T), and the second and fourth position of the second repeat (R2P2, T instead of C, and R2P4, T instead of A). In panels A–C, hydrogen bonds and salt bridges are indicated by dashed grey lines, vdW interactions (distances between 3.3–3.7 Å) by dashed green lines.

*Se*IdeR interacts with DNA in the manner typical for wHTH DNA-binding domains and similarly to other IdeR/DtxR-DNA complexes (22,59). Each IdeR monomer recognizes one of the four five-nucleotide repetitions (CCTAA) conserved in the palindromic 19-bp consensus. The wHTH motif is anchored to the DNA on both edges of the major groove by hydrogen bonds and salt bridges with the sugar-phosphate backbone of the DNA, facilitated by residues from the first helix of the wHTH motif on one side and residues from the second, so-called recognition helix on the other, and the recognition helix is thereby inserted into the major groove. The wing of the wHTH motif interacts with the DNA backbone on the minor groove edge, thus clamping the backbone between the wing and the first helix of the wHTH motif (Figure 4A). Notably, only one direct hydrogen bond is formed between the protein and a DNA base, between Gln43 and the first cytosine of each CCTAA repeat, or guanine at the central G-C basepair of the palindrome (Figure 4A and B, Supplementary Figure S4A). A water-mediated hydrogen bond between Gln43, the backbone carbonyl group of Pro39 and the second cytosine can also be observed in most IdeR subunits and is likely always present. Additionally, van der Waals (vdW) interactions are formed by Ser37 and Pro39 with the thymine in the third position of the repeat and Thr40 with the cytosine in position 2 (Figure 4A and B, Supplementary Figure S4A). The side chains of Ser37 and Thr40 interact with the DNA backbone as well (Figure 4A). The side chain of Pro39 is also in close proximity to the A-T basepair in the fourth position, though these vdW interactions appear to be unspecific (see below; Figure 4A and B). It should be noted that these four residues, Ser37, Pro39, Thr40 and Gln43, and their interactions with DNA bases are conserved in previous structures of IdeR/DtxR-DNA complexes, even if not all of these interactions were discussed in the papers describing them (22,59).

We also determined the crystal structure of the Co^2+^-activated complex of IdeR and C10S1 DNA (Table 1). The structure of this complex does not display any significant differences compared to the complexes with the consensus sequence (Supplementary Figures S1B and S2D, Supplementary Table S3). The C10S1 sequence differs from the consensus in position 3 of the first repeat, and positions 2 and 4 of the second repeat (Table 2). These differences affect the interactions with one subunit of each IdeR dimer. Specifically, the vdW interactions with Pro39 and Ser37 of one IdeR subunit, and the vdW interactions with Pro39 and Thr40 of the IdeR subunit bound to the neighboring major groove are affected. However, the only notable differences regarding these distance-dependent interactions are the absence of the thymine methyl group in position 3 of the first repeat and the presence of a thymine methyl group in position 2 of the second repeat, as the distances between Pro39 and the fourth base pair are very similar regardless of the nature of the bases (Figure 4C). The water-mediated hydrogen bond with Glu43 and Pro39 should not be affected by the different base in position 2 of the second repeat, but the water molecule, though likely present, was not clearly observed in the electron density and was not modelled. Despite these differences, IdeR binds to the C10S1 sequence in the same way as to the consensus sequence (Figure 4C).

### DNA sequence variants suggest a reexamination of the role of the base interactions of IdeR

**Figure 5.**
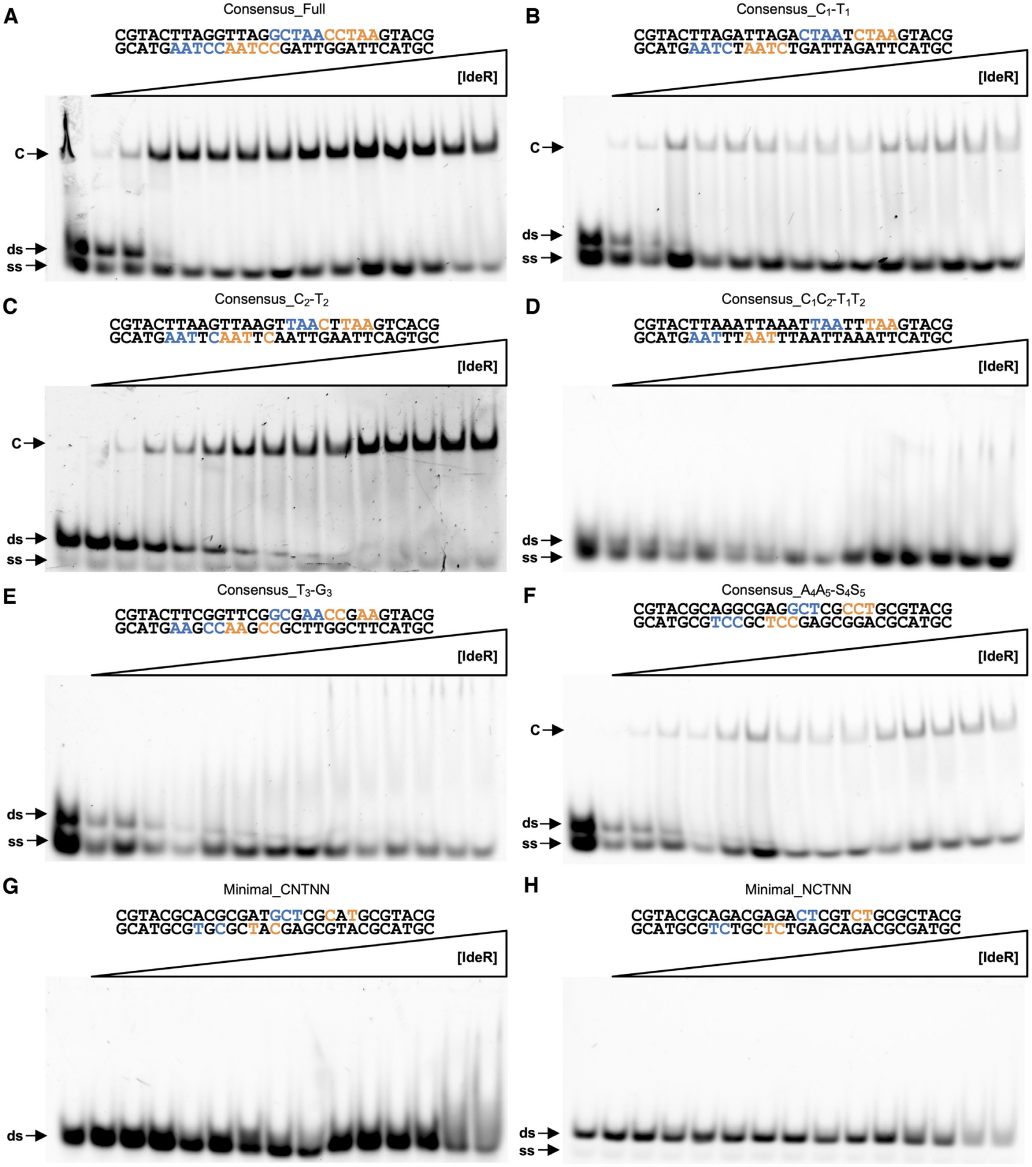
EMSA analysis of the effect of different mutations in the DNA recognition sequence on DNA binding by IdeR. Binding of IdeR^WT^ to (**A**) the full consensus DNA sequence, (**B**) the Consensus_C_1_-T_1_, (**C**) Consensus_C_2_-T_2_, (**D**) Consensus_C_1_C_2_-T_1_T_2_, (**E**) Consensus_T_3_-G_3_, (**F**) Consensus_A_4_A_5_-S_4_S_5_, (**G**) Minimal_CNTNN, and (**H**) Minimal_NCTNN sequences. IdeR was added in increasing concentrations (15 nM–2.25 μM dimer) to 30 nM fluorescence-labeled double-stranded DNA probe in the presence of 30 μM Co^2+^ and competitor DNA. Protein–DNA complexes were resolved on a 4% Tris-acetate polyacrylamide gel. The left-most lane is a control reaction without protein. ds, unbound double-stranded DNA probe; ss, non-hybridized single-stranded DNA; C, protein–dsDNA complex.

To interrogate the DNA recognition mechanism of IdeR and its pattern recognition flexibility, we designed a set of DNA sequences diverging from the 19-bp consensus at different key positions. As shown in Figure 5A, IdeR has a higher affinity (with an apparent *K*_D_ of ∼45 nM, Table 2) for this consensus DNA sequence compared to the native binding sites tested before. The observed differences in affinity confirm the relevance of the mismatches present in the native binding sites, as some of those mismatches are located in the regions that are contacted by the recognition helix of IdeR.

IdeR interacts most strongly with the first and second cytosine of each CCTAA repeat of the recognition sequence, forming hydrogen bonds with both bases and vdW interactions with the second (Figure 4A and B, Supplementary Figure S4A). Based on analysis of the IdeR–DNA complex structures, we reasoned that Gln43 should be able to hydrogen-bond to any base in position 1 of the repeat, although it would have to move to accommodate the methyl group of a thymine in this position (Supplementary Figure S4B). Replacing the cytosine in position 1 with a thymine in every repeat of the recognition sequence (see Consensus_C_1_-T_1_ in Table 2) had no significant effect on IdeR affinity (Figure 5B). IdeR bound to this modified target with an apparent *K*_D_ of ∼70 nM.

The water-mediated hydrogen bond between Gln43 and the base in position 2 should be able to be formed with either pyrimidine, but not with a purine in this position, whereas the Thr40 interaction should be sensitive to all changes in position 2 (Supplementary Figure S4C). Thr40 would have to move to accommodate a thymine methyl group (see Figure 4C), while the distance to a purine in position 2 would be significantly longer. Mutating the cytosine in position 2 to a thymine in every repeat of the consensus sequence (see Consensus_C_2_-T_2_ in Table 2) had no significant effect on IdeR recognition (Figure 5C), causing only a mild decrease in affinity (with an apparent *K*_D_ of ∼105 nM).

However, when both cytosines were simultaneously changed to thymines (see Consensus_C_1_C_2_-T_1_T_2_ in Table 2), we did not observe any specific binding of IdeR (Figure 5D), even when increasing the IdeR concentration 10-fold compared to the conditions tested in all previous EMSAs (Supplementary Figure S5A), implying that at least one of the cytosine bases is required for IdeR recognition.

Next we replaced the thymine in position 3, which forms vdW interactions with Ser37 and Pro39 of IdeR, with a guanine in each CCTAA repeat (see Consensus_T_3_-G_3_ in Table 2), a change which should disrupt these interactions (Supplementary Figure S4D). IdeR was not able to bind specifically to this DNA sequence (Figure 5E). As with the C_1_C_2_-T_1_T_2_ mutation, higher concentrations of IdeR were tested to confirm the absence of specific binding (Supplementary Figure S5B). This result suggests that a thymine in position 3 of the repeat plays a key role in IdeR recognition.

The two remaining adenines of each CCTAA repeat were also mutated to cytosine or guanine (see Consensus_A_4_A_5_-S_4_S_5_ in Table 2). Although these mutations do not affect specific base interactions with IdeR, we observed a drop in IdeR affinity with an estimated apparent *K*_D_ of 128 nM (Figure 5F).

Concluding that each DNA quintet recognized by IdeR requires at minimum a thymine in the third position, and a cytosine in the first or second position, we designed two DNA sequences that should fulfill these minimum base contact requirements for IdeR recognition, but preserve none of the other conserved bases of the recognition sequence (see Minimal_CNTNN and Minimal_NCTNN in Table 2). However, no specific binding of IdeR was observed (Figure 5G and H) even when using high concentrations of IdeR (Supplementary Figure S5C and D). Noting that the number of specific base interactions provided by these sequences should not be different from those of the Consensus_C_2_-T_2_ and Consensus_C_1_-T_1_ targets, respectively, together with the lower affinity observed for the Consensus_A_4_A_5_-S_4_S_5_ target, these results indicate that a sequence-dependent recognition mechanism other than direct specific base interactions plays a key role in target recognition.

### IdeR variants suggest an indirect readout mechanism for specific DNA binding

**Figure 6.**
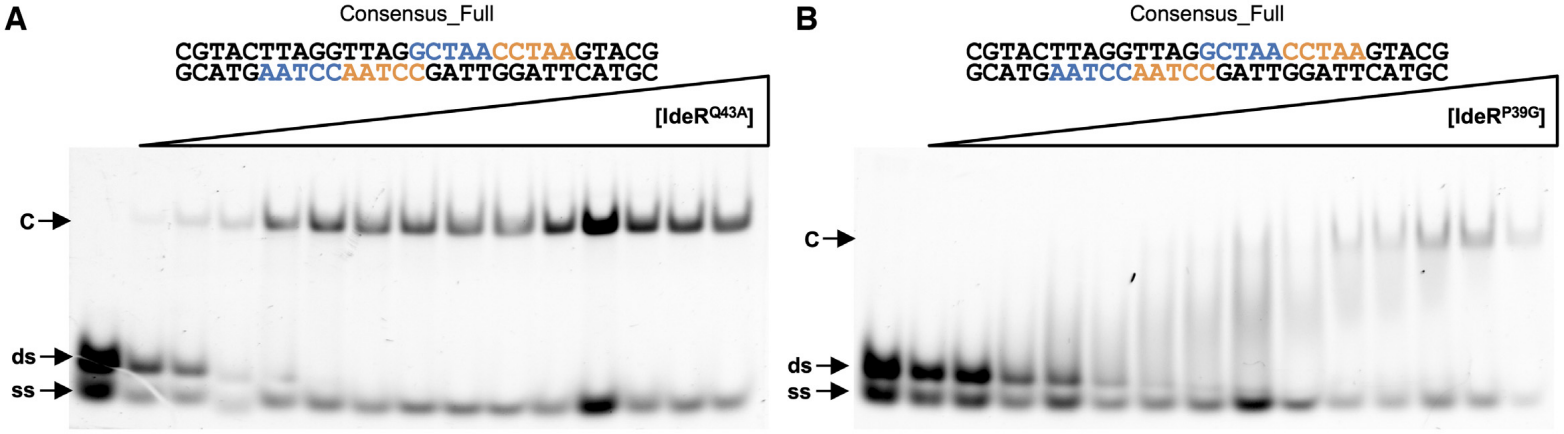
EMSA analysis of the effect of different mutations in the IdeR recognition helix on DNA binding by IdeR. Binding of the (**A**) IdeR^Q43A^ and (**B**) IdeR^P39G^ variants to the consensus DNA sequence. The IdeR variants were added in increasing concentrations (15 nM–2.25 μM dimer) to 30 nM fluorescence-labeled double-stranded DNA probe in the presence of 30 μM Co^2+^ and competitor DNA. Protein–DNA complexes were resolved on a 4% Tris-acetate polyacrylamide gel. The left-most lane is a control reaction without protein. ds, unbound double-stranded DNA probe; ss, non-hybridized single-stranded DNA; C, protein–dsDNA complex.

To clarify the relevance of the base interactions with the recognition helix of IdeR, we generated two IdeR variants, IdeR^Q43A^ and IdeR^P39G^, that should disrupt base interactions while not affecting backbone interactions. When testing the affinity of IdeR^Q43A^ for the consensus sequence with EMSA, we did not observe any significant differences compared to IdeR^WT^ (Figure 6A; apparent *K_D_* ∼65 nM), demonstrating that the hydrogen bonds between Gln43 and the DNA bases are not important for the recognition of the DNA target. Furthermore, these results show that the absence of IdeR^WT^ binding to the sequence Consensus_C_1_C_2_-T_1_T_2_ (Figure 5D) was not due to a disruption of the Gln43-C_1_/C_2_ interactions.

**Figure 7.**
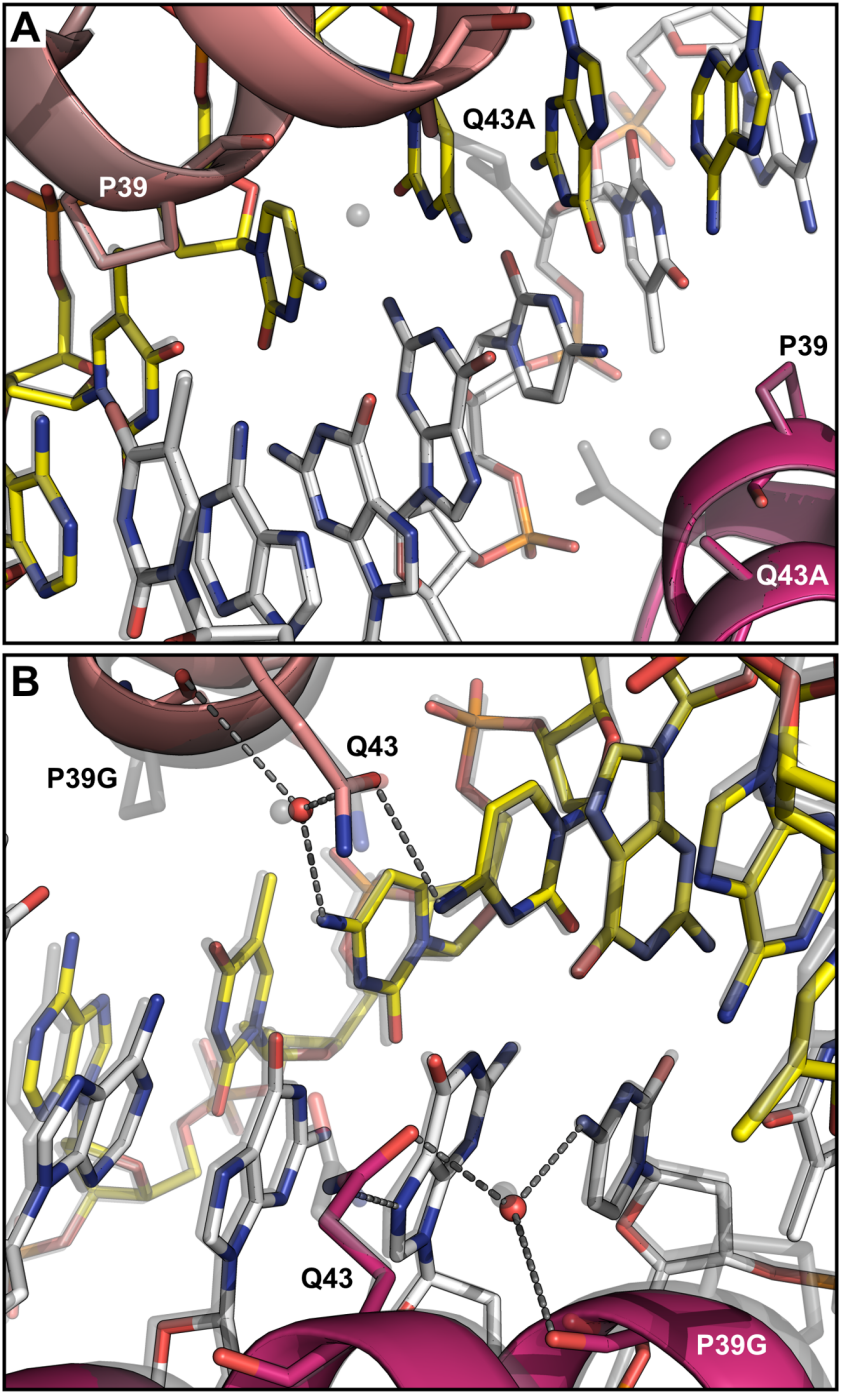
Crystal structures of IdeR variants in complex with the consensus DNA recognition sequence. Interactions between Co^2+^-activated (**A**) IdeR^Q43A^ or (**B**) IdeR^P39G^ and DNA bases, focused on the central G–C basepair in the consensus DNA-binding sequence. The IdeR variants (colored by subunit) are shown superimposed with the Fe^2+^-activated IdeR^WT^-consensus DNA complex structure (transparent grey). In panel B, hydrogen bonds between IdeR^P39G^ and DNA bases are indicated by dashed grey lines.

To discard the possibility that other residues have taken the role of Gln43 in this IdeR variant and established new hydrogen bonds, we obtained the crystal structure of DNA-bound IdeR^Q43A^ (Table 1, Figure 7A, Supplementary Figure S6A–C). The structure is essentially identical to that of the IdeR^WT^-DNA complexes, despite the loss of both the direct hydrogen bond between Gln43 and the first cytosine of each CCTAA repeat as well as the water-mediated hydrogen bond with the second cytosine. It is unclear if the water molecule bound to the second cytosine is lost due to the mutation or not observed as a result of the lower resolution of this structure (Table 1), but it is clearly present in one of the four IdeR^Q43A^ subunits. No additional interactions were observed in the crystal structure of this complex (Figure 7A), corroborating that the base contacts of Gln43 in the recognition helix of IdeR are not required for correct target recognition and binding.

As for the IdeR^P39G^ variant, the EMSA results show specific binding to the consensus target, but the affinity is significantly affected by the mutation, resulting in an apparent *K*_D_ of ∼264 nM (Figure 6B). Due to the special structural features of both proline and glycine residues we cannot conclude whether this loss of affinity is caused by the absence of the vdW interactions with the DNA, or if this protein variant is functionally impaired. However, as IdeR^P39G^ is still able to recognize its target and form a stable complex, we can reason that the absence of IdeR^WT^ binding to the Consensus_T_3_-G_3_ sequence (Figure 5E) was not caused by the disruption of the vdW interactions between Pro39 and the thymine in position 3.

We also obtained the crystal structure of IdeR^P39G^ bound to the consensus DNA to confirm that no other interactions with the essential thymines of the recognition sequence are formed in this IdeR variant (Table 1, Figure 7B, Supplementary Figure S6). Despite the drastic difference between the wild-type and mutated residue, in the DNA-bound state the structure of the recognition helix is essentially unaffected by the mutation. The hydrogen bond formed by the backbone carbonyl group of residue 39 with the water molecule, which is also bound to Gln43 and the cytosine in position 2 of the repeat, is not disrupted by the mutation.

Altogether, our results indicate that IdeR recognizes its targets by an indirect readout mechanism, perceiving the sequence-dependent structure of the DNA instead of or in addition to the chemical signatures of specific DNA bases.

### DNA structure prediction separates IdeR-binding from non-binding sequences

To evaluate if the different DNA sequences that were assessed for binding to IdeR display structural differences that may be recognized by IdeR, we used the DNAshape and DNAphi web servers to predict four structural parameters and the electrostatic potential in the minor groove, respectively, of these DNA sequences (49,50). Interestingly, several of the predicted structural features clearly separate the sequences that are recognized by IdeR from those that are not, and even the high affinity binders from the lower affinity binders (Supplementary Figures S7 and S8). In particular minor groove width and the electrostatic potential in the minor groove stand out as features that differ between binding and weak or non-binding sequences and could therefore be recognized by IdeR.

## DISCUSSION

*Se*IdeR recognizes the 19-bp DNA consensus sequence established for other IdeR/DtxR iron sensors consisting of a palindromic repeat of four CCTAA motifs. Each of these four repeats is recognized by an IdeR monomer with its wHTH DNA-binding motif, resulting in a two-dimer complex with the full DNA sequence. This sequence can be found at 37 regions in the *S. erythraea* genome with different degrees of conservation. The number of targets is consistent with those found in other organisms such as *M. tuberculosis* or *C. diphtheriae*. IdeR binding was confirmed for the siderophore cluster comprised of genes SACE_2689 to SACE_2697 and for the cluster coding for the respiratory chain NADH dehydrogenase Nuo complex I (genes SACE_6902 to SACE_6889).

Complex I of the respiratory chain has been linked to ROS production in mitochondria (16). Little is known about complex I contribution to ROS formation in bacteria, but some species seem to favor the use of complex II in high oxygen conditions, despite the fact that complex II has a bigger role in ROS production in these species (61,62). The *nuo* gene cluster has been shown to be induced by iron in other bacteria such as *M. tuberculosis* and *Geobacter sulfurreducens* (63,64). While no iron-dependent regulator has been identified as responsible for this induction in *M. tuberculosis*, in *G. sulfurreducens* it was found to be under the control of the ferric uptake regulator Fur (63,64). Our results indicate that in *S. erythraea*, complex I production is controlled by IdeR.

The binding of IdeR to the promoter of the *nuo* gene cluster in *S. erythraea* is the first reported example of the formation of an IdeR-DNA complex with only one dimer of the transcriptional regulator. This finding suggests a redefinition of the consensus of the DNA targets for iron-dependent IdeR/DtxR regulators from the 19-bp TTAGGTTAGSCTAACCTAA to the 14-bp TTAGGNNNNCCTAA consensus, which may result in the discovery of new targets in other IdeR/DtxR species. What the function of IdeR at such half sites is remains to be investigated.

While it may be a crystallization artifact, we cannot exclude the possibility that the SH3 domain swap we observe in the IdeR-DNA complex structures is used *in vivo* to cross-link IdeR dimers bound to adjacent binding sites. Since the loop linking the dimerization and SH3-like domains is not resolved in previous crystal structures of DtxR/IdeR-DNA complexes, a similar swap may have occurred in these cases (22,59). A similar function for the SH3-like domain has previously been proposed for the *Streptococcus pyogenes* manganese sensor MtsR which was found to oligomerize on DNA. The interaction between neighboring dimers was shown to be mediated by the SH3-like domain and important for proper gene regulation by MtsR (65). The residues mediating this interaction between the SH3-like domains are highly conserved among the DtxR family manganese sensors (MntR) that possess an SH3-like domain, but not among the iron-sensing family members, suggesting that this oligomerization mechanism is common to MntR proteins, but not used by IdeR/DtxR proteins. In contrast, an inter-molecular interaction of the SH3-like domains was observed in *Mycobacterium smegmatis* IdeR in the inactive metal-free state, whereas the SH3-like domain associated with the N-terminal domains in an intra-molecular manner in the active metal-bound state, thus mediating the metal-dependent activation and inactivation of IdeR (66). It appears that the SH3-like domain is only weakly associated with the N-terminal domains of IdeR and, while clearly important for its function, has evolved different allosteric regulation mechanisms in different organisms. Our structures suggest an avenue for further investigation of its function.

In this work, we show evidence of an indirect readout mechanism for IdeR. Indirect readout is a recognition mechanism based on the structural reading, rather than molecular reading, of a DNA sequence by its DNA-binding protein, which can be mediated by contacts with the minor groove or the DNA backbone (67,68). Although it is well established that indirect readout is a common DNA recognition mechanism among eukaryotic transcription factors, the contribution of this mechanism is frequently overlooked and underestimated for prokaryotic transcription factors, in particular those that exhibit high sequence specificity (67–76). This recognition mechanism was previously proposed for DtxR-like proteins by Lee and Holmes (2000) (51), who considered that the Gln43 interactions with the cytosine bases were not enough to explain the specificity of this transcription factor. However, Chen *et al.* (77) described the vdW interactions with the thymine bases, which would theoretically add to the Gln43 interactions to support a direct readout of the DNA sequence for this type of proteins. Here we show that neither of these interactions determines the recognition process, and that an indirect readout mechanism is required to explain DNA recognition by IdeR.

It might be argued that the vdW interactions with the thymine bases, mediated by Pro39 and Ser37, are still relevant for DNA recognition, as IdeR does not recognize a sequence lacking the thymines, and the P39G variant did not show the same affinity for the consensus sequence as IdeR^WT^. However, several pieces of evidence suggest that these interactions are non-essential: (i) considering that the Gln43 interactions are not required for recognition, and that IdeR does not recognize a DNA sequence that provides only the specific vdW contacts (Minimal_NCTNN), the strength of the vdW interactions does not suffice to account for specific recognition of the full target sequence; (ii) the IdeR^P39G^ variant is still able to specifically recognize its DNA target, implying a recognition mechanism independent of these vdW interactions; (iii) the loss of affinity of this IdeR variant may be caused by the loss of the specific and/or unspecific vdW interactions between Pro39 and the DNA; (iv) the loss of affinity may also be caused by the biochemical nature of the exchanged residues that might result in undesired effects on the dynamics of the protein, affecting the flexibility of the wHTH motif, and thereby the affinity for its DNA target; (v) as shown by the lack of binding of IdeR to the Minimal_CNTNN and Minimal_NCTNN DNA sequences, the presence of all thymine bases involved in the vdW interactions is not enough for target recognition; (vi) the work done by Chen *et al.* (77) and Spiering et al. (56), although highlighting the relevance of the thymine bases for recognition, showed binding of two dimers to a DNA sequence lacking both thymine bases for one of those dimers, again implying a recognition mechanism that is independent of the vdW interactions with the thymines.

The cooperativity observed for the interaction between IdeR and its DNA targets lends support to the proposed indirect DNA recognition mechanism. The absence of one-dimer complexes in any of the EMSAs performed with all full-target DNA sequences indicates that the binding of both IdeR dimers to the full 4-repeat recognition sequence is cooperative. We observe no direct interaction between these two protein dimers in the crystal structures. The closest contact between the two DNA-bound IdeR dimers is formed by the Gln43 residues interacting with the central G-C base pair of the palindrome, which approach each other to within ∼4.8 Å (Figure 4C). However, we note that no loss of cooperativity is observed for IdeR^Q43A^ (Figure 6A), thus ruling out that this residue is responsible for cooperative DNA binding. The cooperativity must therefore arise from a structural change of the DNA double helix upon binding of the first IdeR dimer. Along with indirect readout, DNA-mediated allostery is increasingly recognized as an important factor in the cooperative assembly of DNA-binding proteins on DNA (72,78–84). However, in most cases of DNA-mediated allostery observed to date, eukaryotic genomes are concerned, where pairs of different transcription factors commonly bind cooperatively to juxtaposed or overlapping binding sites, and large complexes of multiple different transcription factors (enhanceosomes) can be formed at some sites.

In support of the hypothesis that the DNA mediates cooperativity between IdeR dimers, a comparison of the DNA conformation observed in the IdeR complex crystal structures with the predicted structural parameters for these sequences indicates that the naked DNA adopts a different conformation (Supplementary Figure S9). The observed and predicted minor groove width in particular is very different. Specifically, the minor groove is predicted to be wide at the TpA steps in the naked consensus DNA, but in contrast is very narrow at these steps in the protein-DNA complexes. This is not unexpected given that out of all base pair steps, the TpA step has the weakest stacking interaction, leading to a widening of the minor groove at these steps in naked DNA, but also allowing the minor groove around TpA steps to be easily deformed by protein binding (67). As the prediction algorithm has been extensively validated (49,85), the discrepancies are likely due to distortions caused by IdeR binding and/or crystal packing interactions in the crystal structures. Since the DNA strands are not involved in crystal contacts in our structures and should thus not be constrained by anything other than the bound protein, we consider it most likely that the differences between the observed DNA conformation in the IdeR complexes and the predicted DNA conformation in the absence of protein are indeed caused by IdeR binding. Nevertheless, solution structures of the different DNA sequences, in the free as well as the IdeR-bound state, will be required to evaluate their conformational differences.

In conclusion, we show that IdeR recognizes its targets by reading the sequence-dependent DNA backbone structure instead of or in addition to reading specific base signatures. The similarities of the wHTH motifs of most iron-dependent IdeR/DtxR regulators, in line with the fact that most IdeR/DtxR proteins recognize the same consensus sequence, suggest that they use the same DNA recognition mechanism. Future work will determine to which extent indirect readout contributes to target site recognition in other bacterial transcriptional regulators, including the DtxR family manganese-dependent transcriptional regulators which are more structurallly and functionally divergent from IdeR.

## DATA AVAILABILITY

Atomic coordinates and structure factors for the reported crystal structures have been deposited with the Protein Data Bank under accession numbers 7B1V (Co^2+^-activated IdeR^WT^), 7B1Y (Co^2+^-activated IdeR^WT^ in complex with consensus DNA), 7B20 (Fe^2+^-activated IdeR^WT^ in complex with consensus DNA), 7B23 (Co^2+^-activated IdeR^WT^ in complex with C10S1 DNA), 7B25 (Co^2+^-activated IdeR^Q43A^ in complex with consensus DNA) and 7B24 (Co^2+^-activated IdeR^P39G^ in complex with consensus DNA).

## Supplementary Material

gkab711_Supplemental_Files

## Notes

Present address: Francisco Javier Marcos-Torres, Max Planck Institute for Terrestrial Microbiology, Karl-von-Frisch Str. 10, DE-35043 Marburg, Germany.
