# Supplementary material for: The bacterial iron sensor IdeR recognizes its DNA targets by indirect readout
Source: Nucleic Acids Res. 2021 Aug 20;49(17):10120–35. doi: 10.1093/nar/gkab711 (PMC8464063; doi:10.1093/nar/gkab711)
Supplement: gkab711_Supplemental_Files [file gkab711_supplemental_files.zip › IdeR_SI.pdf]

Supplementary Information for

The bacterial iron sensor IdeR recognizes its DNA targets by indirect readout

Francisco Javier Marcos-Torres[1,†], Dirk Maurer[1,†], Linda Juniar[1] and Julia J. Griese[1,*]

[1] Department of Cell and Molecular Biology, Uppsala University, SE-751 24 Uppsala, Sweden

[†] These authors contributed equally to this work.

* To whom correspondence should be addressed. Tel: +46-18 471 4982; Email: julia.griese@icm.uu.se

Present Address: Francisco Javier Marcos-Torres, Max Planck Institute for Terrestrial Microbiology, Karl-von-Frisch Str. 10, DE-35043 Marburg, Germany

Contents

Supplementary Figures

Figure S1: Comparison of different IdeR^{WT} complex structures.

Figure S2: The metal-binding sites in the different IdeR^{WT} complex structures.

Figure S3: Orientation of the DNA and domain swap in crystals of IdeR-DNA complexes.

Figure S4: Two-dimensional representation of the specific interactions between IdeR and DNA bases in the consensus DNA recognition sequence and possible interactions with variations of the consensus sequence.

Figure S5: Additional EMSA analysis of the effect of different mutations in the DNA recognition sequence on DNA binding by IdeR.

Figure S6: Crystal structures of IdeR variants in complex with the consensus DNA recognition sequence.

Figure S7: Structural features predicted for the variations of the IdeR consensus DNA recognition sequence which were assessed for binding to IdeR.

Figure S8: Structural features predicted for the wild-type *S. erythraea* promoter sequences as well as the full and half-site consensus DNA recognition sequences which were assessed for binding to IdeR.

Figure S9: Comparison of the predicted structural features of the naked IdeR consensus DNA recognition sequence with the observed features in the crystal structure of the Fe^{2+}-activated IdeR-consensus DNA complex.

Supplementary Tables

Table S1: List of oligonucleotides used in this study.

Table S3: Root-mean square distance (RMSD) between aligned Cα atoms of different IdeR structures.

Supplementary References

Supplementary Figures

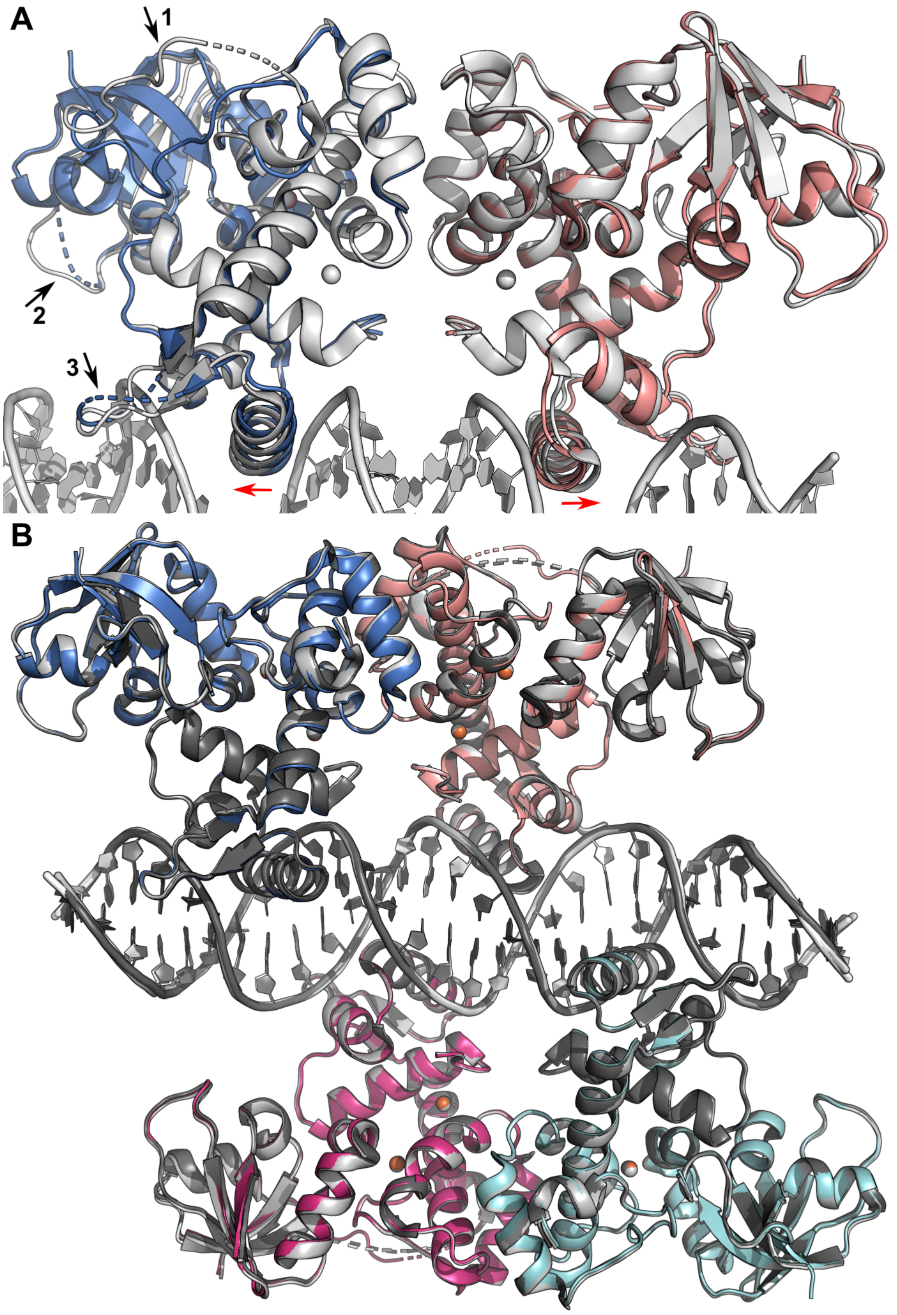

Figure S1. Comparison of different IdeR^{WT} complex structures. (**A**) Overall structure of the Co^{2+}-activated IdeR dimer without DNA, compared to Fe^{2+}-activated IdeR in complex with the consensus DNA-binding sequence. The DNA-free IdeR dimer is shown colored by subunit, superimposed with the Fe^{2+}-activated IdeR-consensus DNA complex (light grey). Metal ions are shown as spheres. DNA binding causes a slight reorientation of the recognition helices (indicated by red arrows), which is required to allow the helices to insert into the major grooves of the DNA. In the DNA-bound state, several loops also assume a different or more ordered conformation (indicated by black arrows): (1) the loop connecting the dimerization and SH3-like domains in both subunits, (2) a long loop in the SH3-like domain in one subunit, and (3) the loop in the wing of the winged HTH motif in the same subunit. (**B**) Superposition of the Fe^{2+}-activated IdeR-consensus DNA complex (colored by subunit) with the Co^{2+}-activated IdeR complexes with the consensus DNA-binding sequence (light grey) and the C10S1 sequence (dark grey).

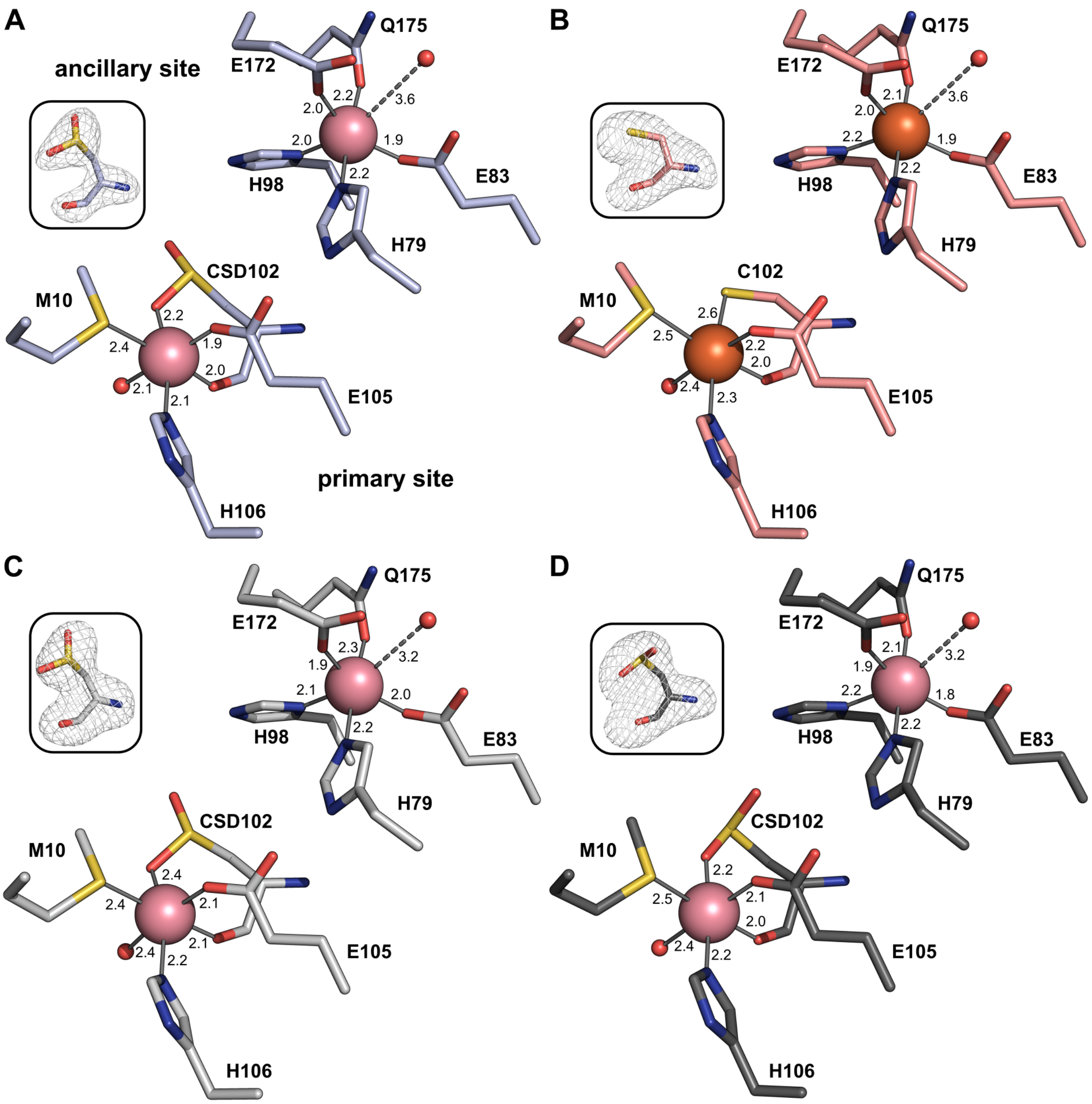

Figure S2. The metal-binding sites in the different IdeR^{WT} complex structures. The metal-binding sites in (**A**) subunit B of the Co^{2+}-activated IdeR dimer without DNA, (**B**) IdeR subunit B in the Fe^{2+}-activated IdeR-consensus DNA complex, (**C**) IdeR subunit D in the Co^{2+}-activated IdeR-consensus DNA complex and (**D**) IdeR subunit B in the Co^{2+}-activated IdeR-C10S1 DNA complex. At the resolution of these structures, no significant differences between the complexes with the physiological activator Fe^{2+} and the mimic Co^{2+} can be discerned. Oxidation of the primary site ligand Cys102 due to radiation damage was observed to varying degrees in the structures described in this study. If oxidation had occurred to a significant degree, the ligand was modelled as *S*-sulfinocysteine (CSD). Metal-ligand bonds are indicated by grey lines, the dashed line between the ancillary site metal ion and water ligand indicating a long, weak bond. Bond distances are given in Å. Insets show mF_o-DF_c omit electron density for C102/CSD102 as grey mesh contoured at +4 σ.

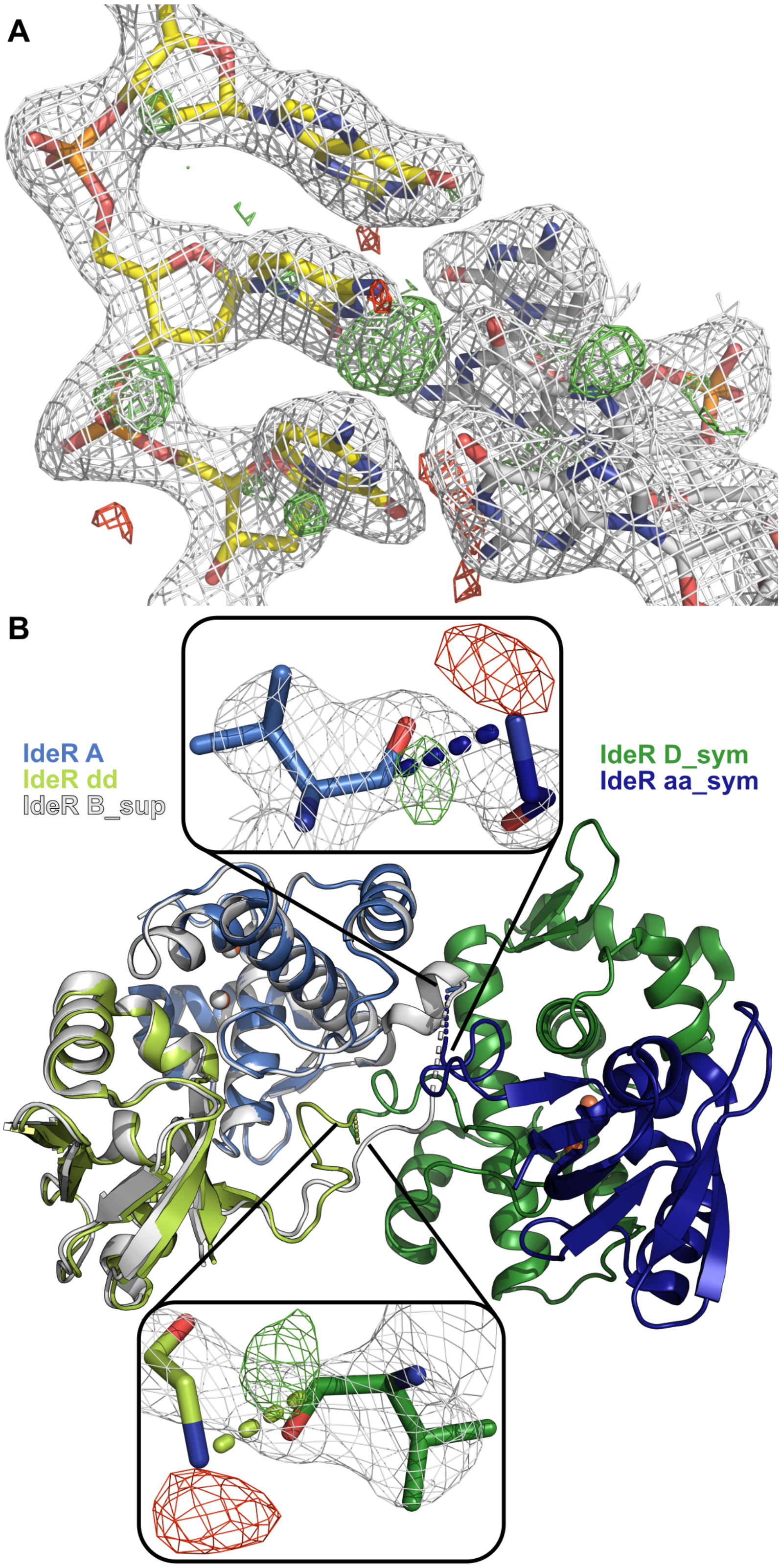

Figure S3. Orientation of the DNA and domain swap in crystals of IdeR-DNA complexes. (**A**) Due to the palindromic nature of the consensus DNA sequence, crystals of IdeR-consensus DNA complexes contain the DNA double helix in both orientations, as evidenced by the ambiguous $2mF_o\text{-}DF_c$ electron density as well as the difference density at the central G-C basepair, in contrast to the well-defined electron density for the surrounding basepairs in the Fe^{2+}-activated IdeR-consensus DNA complex. $2mF_o\text{-}DF_c$ electron density is shown as grey mesh, contoured at 2 σ, $mF_o\text{-}DF_c$ density is shown as green mesh at +3 σ and red mesh at –3 σ. The electron density for the 3 basepairs at each end of the double helix, where the palindrome is broken, is also weaker and more ambiguous (not shown). (**B**) The SH3-like domains of one subunit of each IdeR dimer in the DNA complexes are swapped with a symmetry-related chain. Due to crystal packing interactions, this domain swap is possible for IdeR chains A and D, but not chains B and C. This is illustrated here on the Fe^{2+}-activated IdeR-consensus DNA complex. The SH3-like domain of IdeR subunit A (chain aa_sym, dark blue) associates with the DNA-binding and dimerization domains of subunit D from a symmetry mate (chain D_sym, dark green), and the SH3-like domain originating from subunit D of that symmetry mate (chain dd, lime green) in turn associates with the DNA-binding and dimerization domains of subunit A (chain A, marine). IdeR subunit B (light grey), which does not undergo a domain swap and in which the loop connecting the dimerization and SH3-like domains adopts a different conformation, is shown superimposed on chains A and dd. The connections of the A and aa_sym or D and dd_sym chains are indicated by dashed lines (dark blue and lime green, respectively). Insets show $2mF_o\text{-}DF_c$ electron density for the two residues on either side of the asymmetric unit border, V140 and G141, as grey mesh, contoured at 1.5 σ, and $mF_o\text{-}DF_c$ difference density as green mesh at +3 σ and red mesh at –3 σ.

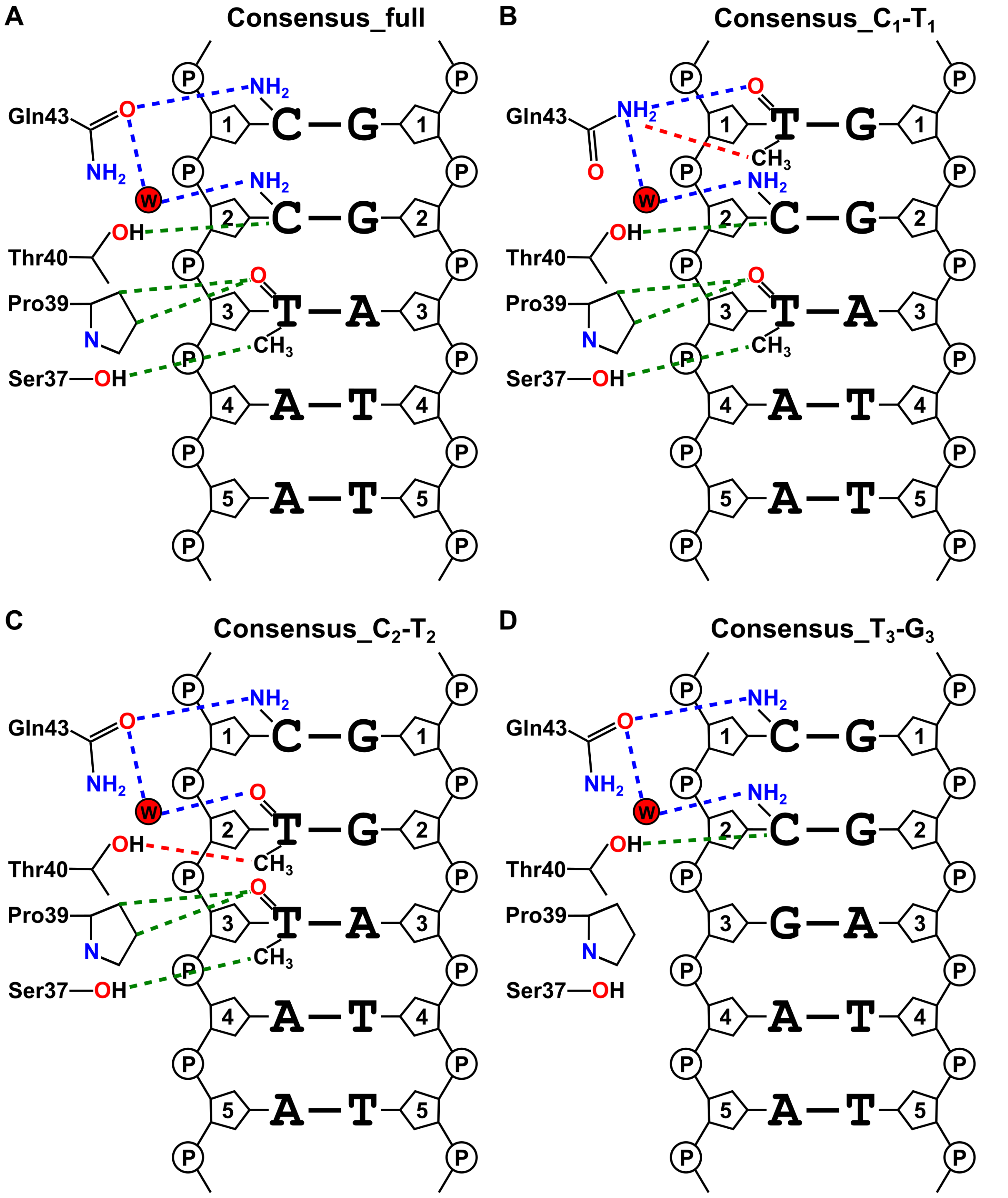

Figure S4. Two-dimensional representation of the specific interactions between IdeR and DNA bases in the consensus DNA recognition sequence and possible interactions with variations of the consensus sequence. (**A**) Specific interactions between IdeR^{WT} and the CCTAA repeat of the consensus DNA recognition sequence observed in the IdeR-consensus DNA complex structures. (**B-D**) Possible interactions of IdeR^{WT} with (**B**) the Consensus_C_1-T_1, (**C**) the Consensus C_2-T_2 and (**D**) the Consensus_T_3-G_3 sequences. Hydrogen bonds are shown as dashed blue lines, vdW interactions as dashed green lines, and potential clashes as dashed red lines.

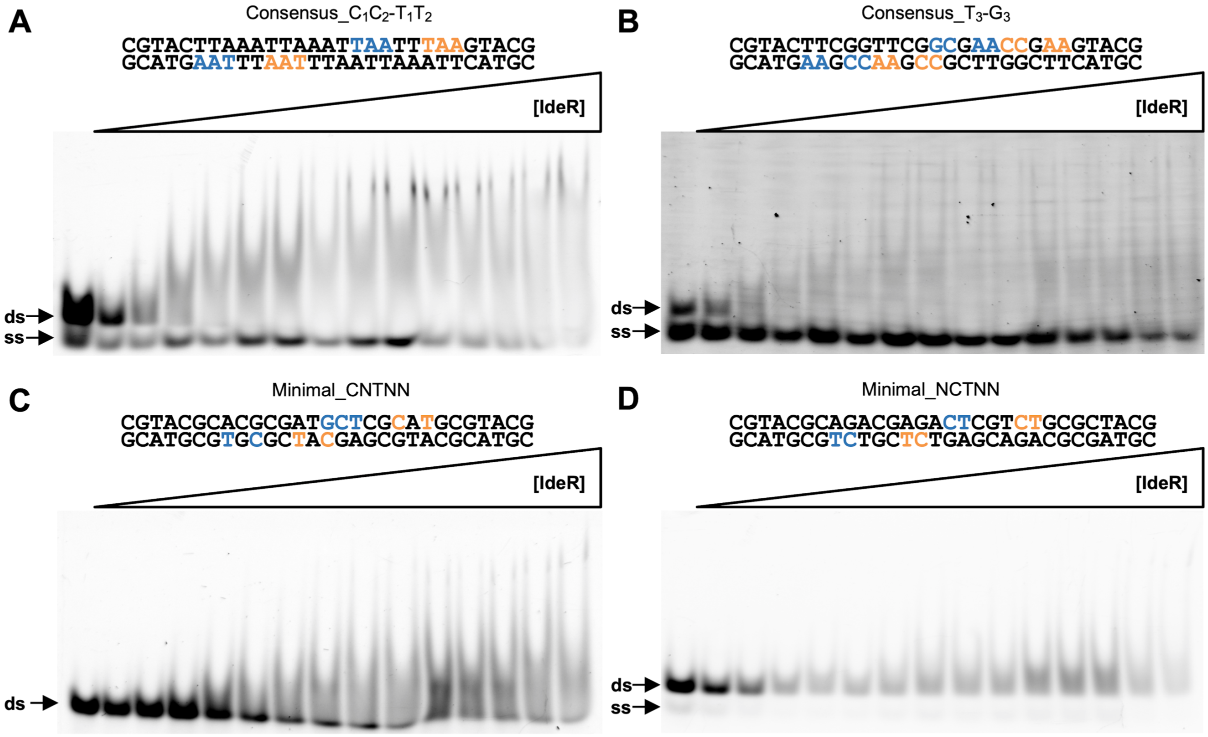

Figure S5. Additional EMSA analysis of the effect of different mutations in the DNA recognition sequence on DNA binding by IdeR. Binding of $IdeR^{WT}$ to the (**A**) Consensus_C_1C_2-T_1T_2, (**B**) Consensus_T_3-G_3 , (**C**) Minimal_CNTNN, and (**D**) Minimal_NCTNN sequences. IdeR was added in increasing concentrations (150 nM – 22.5 μM dimer) to 30 nM fluorescence-labeled double-stranded DNA probe in the presence of 30 μM Co^{2+} and competitor DNA. Note that the IdeR concentrations used in panels A-D are 10-fold higher compared to Figure 5 panels D, E, G and H. Protein-DNA complexes were resolved on a 4% Tris-acetate polyacrylamide gel. The left-most lane is a control reaction without protein. ds, unbound double-stranded DNA probe; ss, non-hybridized single-stranded DNA.

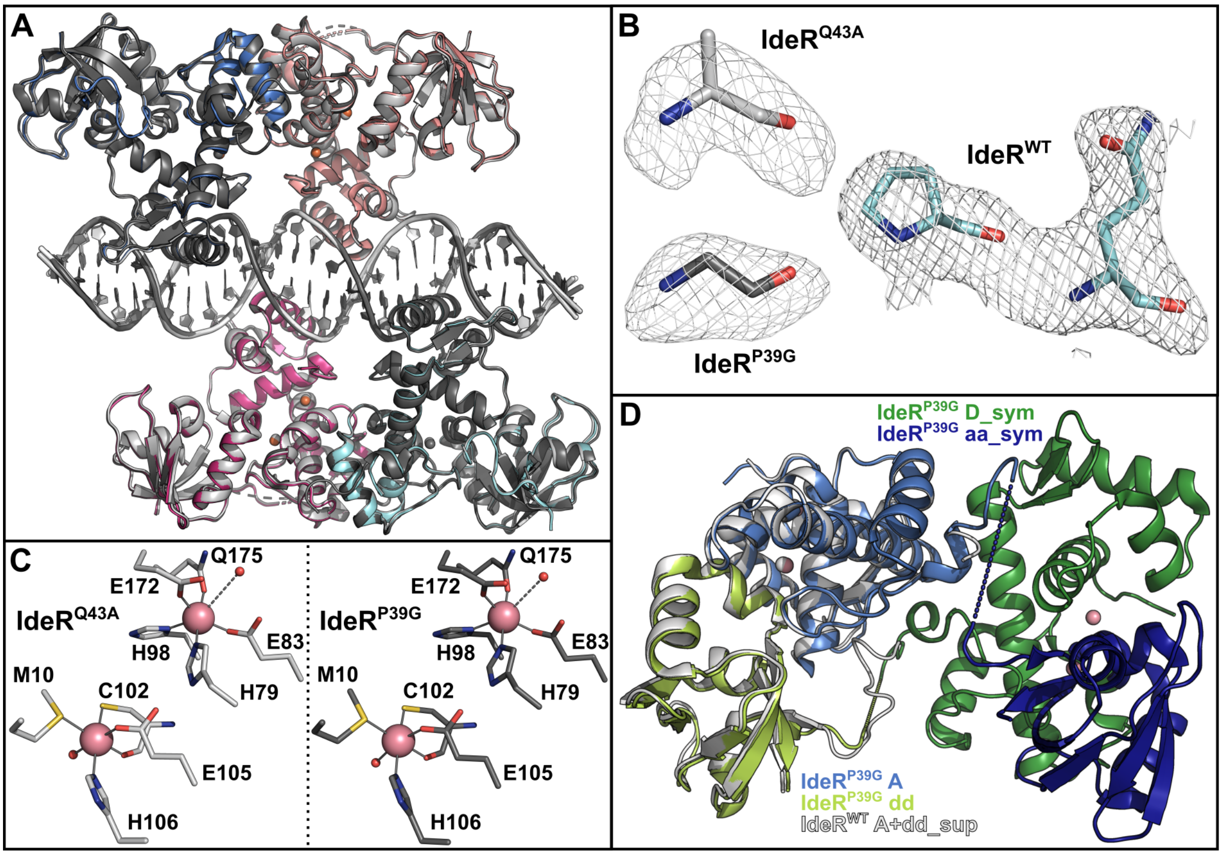

Figure S6. Crystal structures of IdeR variants in complex with the consensus DNA recognition sequence. (**A**) Superposition of the Co^{2+}-activated complexes of IdeR^{Q43A} (light grey) and IdeR^{P39G} (dark grey) and the Fe^{2+}-activated IdeR^{WT} complex (colored by chain) with the consensus DNA-binding sequence. (**B**) mF_o-DF_c omit electron density for A43 in subunit A of IdeR^{Q43A} (light grey) and G39 in subunit D of IdeR^{P39G} (dark grey), compared to mF_o-DF_c omit electron density for P39 and Q43 in subunit D of the Fe^{2+}-activated IdeR^{WT}-consensus DNA complex (cyan), shown as grey mesh contoured at +3 σ. (**C**) The metal-binding sites in subunit B of Co^{2+}-activated IdeR^{Q43A} (light grey, left panel) and subunit B of Co^{2+}-activated IdeR^{P39G} (dark grey, right panel). Oxidation of the primary site ligand Cys102 has not occurred to a significant degree in either crystal. Metal-ligand bonds are indicated by grey lines, the dashed line between the ancillary site metal ion and water ligand indicating a long, weak bond. (**D**) The SH3-like domain swap takes place in IdeR^{P39G}, but the loop connecting the dimerization domain and the swapped SH3-like domain assumes a different conformation than in the other IdeR-DNA complexes. Shown are IdeR^{P39G} chains A (marine) and D_sym (dark green) of the symmetry mate which swaps the SH3-like domain with chain A, each consisting of the DNA-binding and dimerization domains, and the associated swapped SH3-like domains (chain dd, lime green, and chain aa_sym, dark blue), superimposed with the corresponding IdeR^{WT} chains A and dd (light grey) in the Fe^{2+}-activated consensus-DNA complex. The connections of the A and aa_sym or D and dd_sym chains are indicated by dashed lines (dark blue and lime green, respectively).

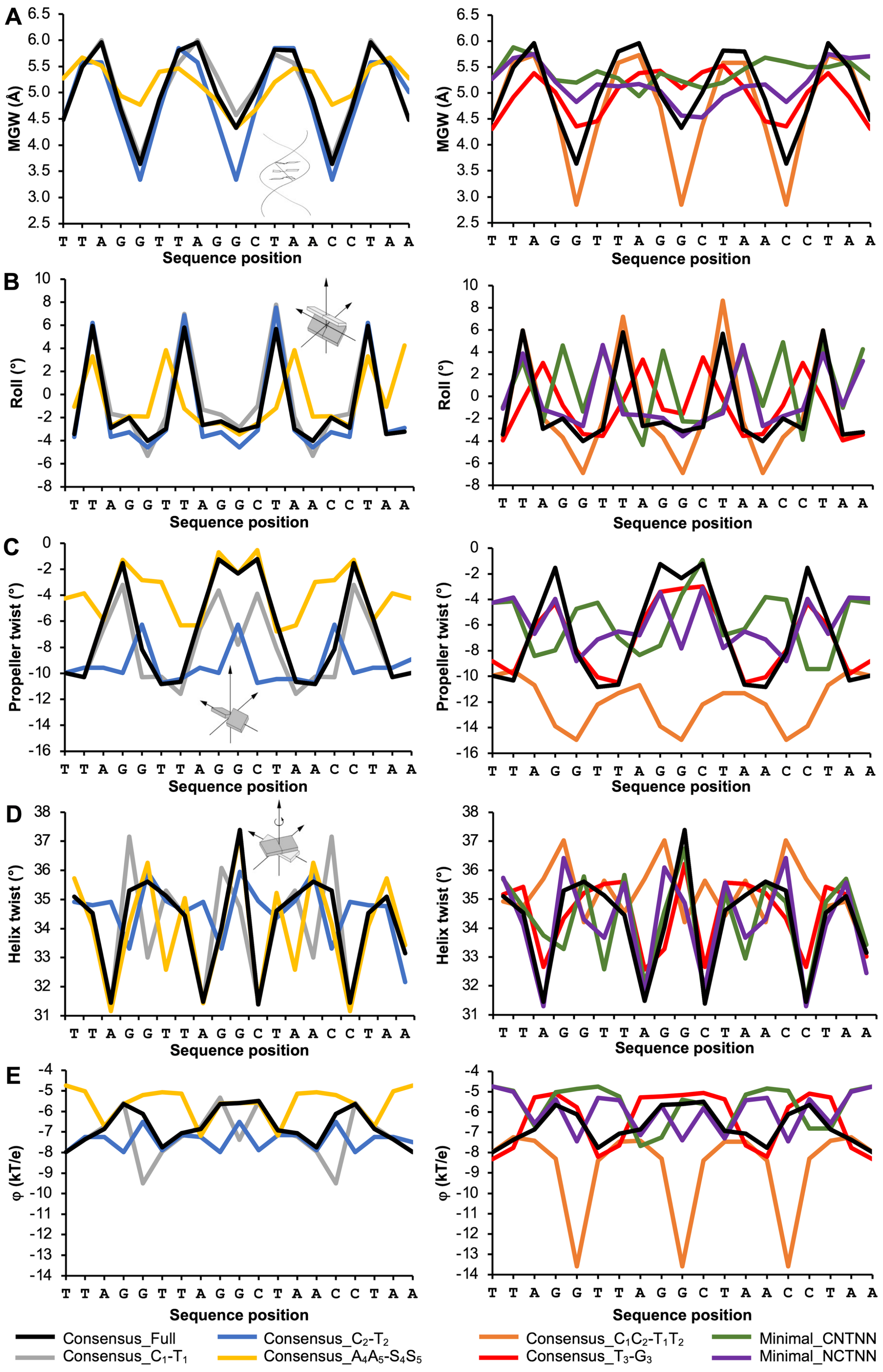

A
B
C
D
E
MGW (Å)
Roll (°)
Propeller twist (°)
Helix twist (°)
φ (kT/e)
T T A G G T T A G G C T A A C C T A A
Sequence position
Consensus_Full
Consensus_C_1-T_1
Consensus_C_2-T_2
Consensus_A_4A_5-S_4S_5
Consensus_C_1C_2-T_1T_2
Consensus_T_3-G_3
Minimal_CNTNN
Minimal_NCTNN

Figure S7. Structural features predicted for the variations of the IdeR consensus DNA recognition sequence which were assessed for binding to IdeR. (**A**) Minor groove width (MGW), (**B**) roll, (**C**) propeller twist, (**D**) helix twist and (**E**) minor groove electrostatic potential φ. The left and right panels show the predictions for binding and non-binding sequences, respectively, while the prediction for the full consensus sequence is included in all panels for easier comparison. The full consensus recognition sequence is shown on the X-axis to indicate the sequence position. Schematic representations of the predicted structural features are shown (1). These features were predicted using the DNAshape and DNAphi web servers (1, 2). Predictions were performed for the full sequences used in the assays, but only the values for the central 19-bp palindrome recognized by IdeR are plotted here. See Table 2 in the main text for the sequences analyzed. All features except for helix twist show a correlation with the affinity of the respective sequence for IdeR. (See also Figure S8.)

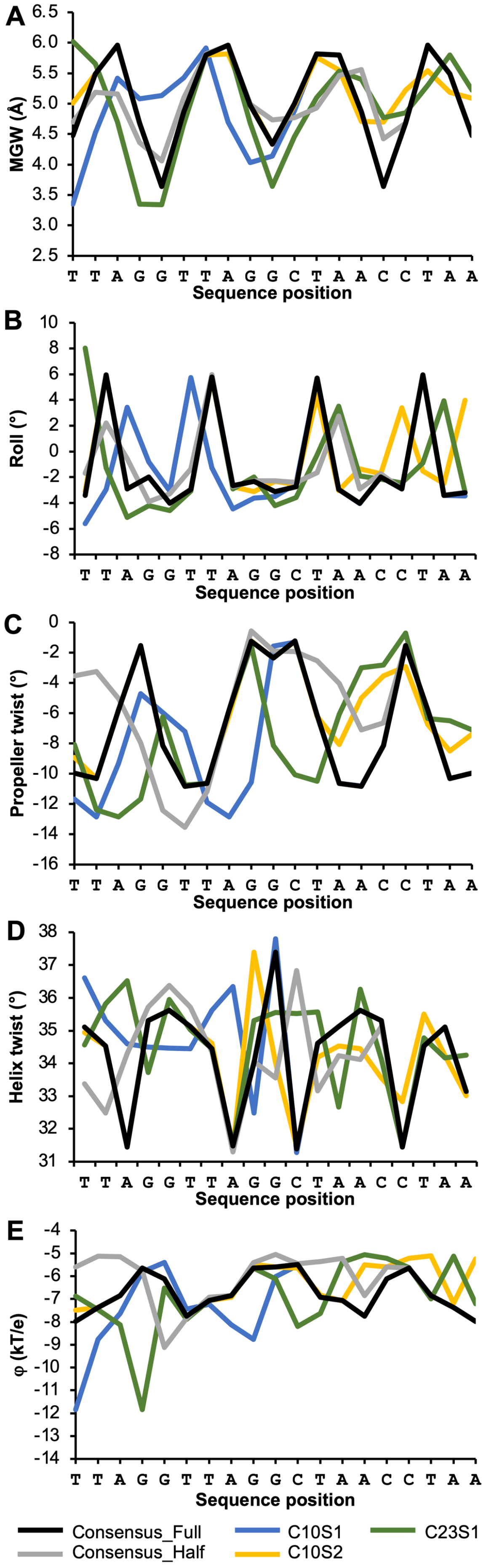

Figure S8. Structural features predicted for the wild-type *S. erythraea* promoter sequences as well as the full and half-site consensus DNA recognition sequences which were assessed for binding to IdeR. (**A**) Minor groove width (MGW), (**B**) roll, (**C**) propeller twist, (**D**) helix twist and (**E**) minor groove electrostatic potential φ. The full consensus recognition sequence is shown on the X-axis to indicate the sequence position. These features were predicted using the DNAshape and DNAphi web servers (1, 2). Predictions were performed for the full sequences used in the assays, but only the values for the central 19-bp palindrome recognized by IdeR are plotted here. See Table 2 in the main text for the sequences analyzed.

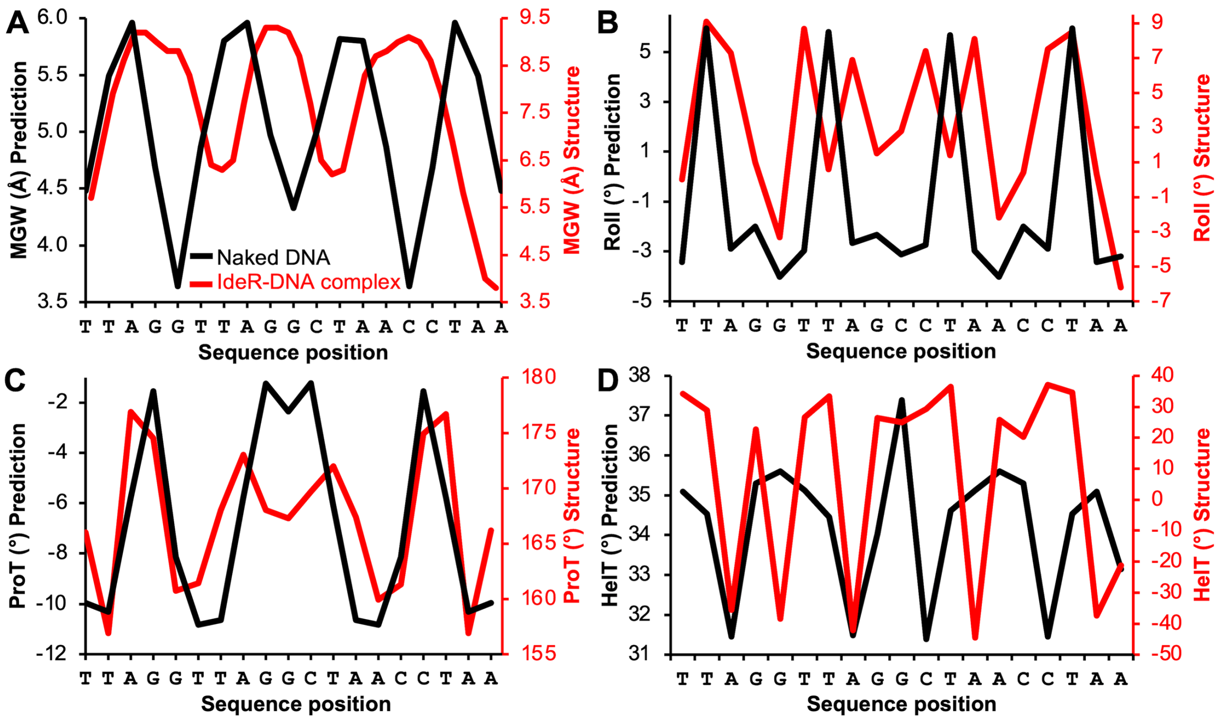

Figure S9. Comparison of the predicted structural features of the naked IdeR consensus DNA recognition sequence with the observed features in the crystal structure of the Fe^{2+}-activated IdeR-consensus DNA complex. (**A**) Minor groove width (MGW), (**B**) roll, (**C**) propeller twist (ProT), (**D**) helix twist (HelT). The consensus recognition sequence is shown on the X-axis to indicate the sequence position. These features were predicted using the DNAshape web server (1), and the crystal structure was analyzed with the Curves+ web server (3, 4). The prediction and analysis were performed for the full sequence used, but only the values for the central 19-bp palindrome recognized by IdeR are plotted here. It should be noted that the definitions of these structural features differ to some extent between DNAshape and Curves+. Therefore different Y-axes are used, and only the trends should be compared.

Supplementary Tables

Table S1. List of oligonucleotides used in this study.

Name	Sequence 5'→3'	Description
IdeR_NdeI_fwd	TATCATATGAACGATCTCATCGATACCACCGAGATG	Forward primer for cloning the *S. erythraea ideR* gene into pET-28a-TEV (*Nde*I restriction site underlined)
IdeR_Hind_stop_ rev	TATAAGCTTTCACTTGACGCGCACCATGACCG	Reverse primer for cloning the *S. erythraea ideR* gene into pET-28a-TEV (*Hind*III restriction site underlined)
IdeR_Q43A_fwd	GAGCGGCCCCACGGTGAGCGCGACGGTC	Forward mutagenic primer to create the Q43A mutation in *S. erythraea ideR* inserted into pET-28a-TEV (mutated nucleotides underlined)
IdeR_Q43A_rev	CTCCATCCGCGCGACCGTCGCGCTCACC	Reverse mutagenic primer to create the Q43A mutation in *S. erythraea ideR* inserted into pET-28a-TEV (mutated nucleotides underlined)
IdeR_P39G_fwd	GGAGCAGAGCGGCGGCACGGTGAGCCAG	Forward mutagenic primer to create the P39G mutation in *S. erythraea ideR* inserted into pET-28a-TEV (mutated nucleotides underlined)
IdeR_P39G_rev	CTGGCTCACCGTGCCGCCGCTCTGCTCC	Reverse mutagenic primer to create the P39G mutation in *S. erythraea ideR* inserted into pET-28a-TEV (mutated nucleotides underlined)
C10S1_FAM_fwd	[FAM]-CGTACTTTGGTAAAGCTAACCTAAGTCACC	Forward strand of the cluster 10 site 1 sequence used for EMSA analyses (5' FAM label)
C10S1_rev	GGTGACTTAGGTTAGCTTTACCAAAGTACG	Reverse strand of the cluster 10 site 1 sequence used for EMSA analyses
C10S2_Cy5_fwd	[Cy5]-GATGCTTAGGTTAGCCTACCCGACGCTGA	Forward strand of the cluster 10 site 2 sequence used for EMSA analyses (5' Cy5 label)
C10S2_rev	TCAGCGTCGGGTAGGCTAACCTAAGCATC	Reverse strand of the cluster 10 site 2 sequence used for EMSA analyses
C23S1_Cy5_fwd	[Cy5]-AGTCATAAAGTTAGGTTCGCCTCACTACT	Forward strand of the cluster 23 sequence used for EMSA analyses (5' Cy5 label)
C23S1_rev	AGTAGTGAGGCGAACCTAACTTTATGACT	Reverse strand of the cluster 23 sequence used for EMSA analyses
Half_Cy5_fwd	[Cy5]-CGTACCCGGTTTAGGGCCACCTAAGTACG	Forward strand of the half site sequence used for EMSA analyses (5' Cy5 label)
Half_rev	CGTACTTAGGTGGCCCTAAACCGGGTACG	Reverse strand of the half site sequence used for EMSA analyses
Cons_Cy5_ fwd	[Cy5]-CGTACTTAGGTTAGGCTAACCTAAGTACG	Forward strand of the Consensus_Full sequence used for EMSA analyses (5' Cy5 label)
Cons_FAM_fwd	[FAM]-CGTACTTAGGTTAGGCTAACCTAAGTACG	Forward strand of the Consensus_Full sequence used for EMSA analyses (5' FAM label)
Cons_rev	CGTACTTAGGTTAGCCTAACCTAAGTACG	Reverse strand of the Consensus_Full sequence used for EMSA analyses
C1-T1_Cy5_fwd	[Cy5]-CGTACTTAGATTAGACTAATCTAAGTACG	Forward strand of the Consensus_C_1-T_1 sequence used for EMSA analyses (5' Cy5 label)
C1-T1_rev	CGTACTTAGATTAGTCTAATCTAAGTACG	Reverse strand of the Consensus_C_1-T_1 sequence used for EMSA analyses
C2-T2_FAM_fwd	[FAM]-CGTACTTAAGTTAAGTTAACTTAAGTCACG	Forward strand of the Consensus_C_2-T_2 sequence used for EMSA analyses (5' FAM label)

Table S1 continued.

Name	Sequence 5'→3'	Description
C2-T2_rev	CGTGACTTAAGTTAACTTAACTTAAGTACG	Reverse strand of the Consensus_C2-T2 sequence used for EMSA analyses
C1C2-T1T2_Cy5_fwd	[Cy5]-CGTACTTAAATTAAATTAATTTAAGTACG	Forward strand of the Consensus_C1C2-T1T2 sequence used for EMSA analyses (5' Cy5 label)
C1C2-T1T2_rev	CGTACTTAAATTAATTTAATTTAAGTACG	Reverse strand of the Consensus_C1C2-T1T2 sequence used for EMSA analyses
T3-G3_Cy5_fwd	[Cy5]-CGTACTTCGGTTCGGCGAACCGAAGTACG	Forward strand of the Consensus_T3-G3 sequence used for EMSA analyses (5' Cy5 label)
T3-G3_rev	CGTACTTCGGTTCGCCGAACCGAAGTACG	Reverse strand of the Consensus_T3-G3 sequence used for EMSA analyses
A4A5-S4S5_Cy5_fwd	[Cy5]-CGTACGCAGGCGAGGCTCGCCTGCGTACG	Forward strand of the Consensus_A_4A_5-S_4S_5 sequence used for EMSA analyses (5' Cy5 label)
A4A5-S4S5_rev	CGTACGCAGGCGAGCCTCGCCTGCGTACG	Reverse strand of the Consensus_A_4A_5-S_4S_5 sequence used for EMSA analyses
CNTNN_Cy5_fwd	[Cy5]-CGTACGCACGCGATGCTCGCATCCGTACG	Forward strand of the Minimal_CNTNN sequence used for EMSA analyses (5' Cy5 label)
CNTNN_rev	CGTACGGATGCGAGCATCGCGTGCGTACG	Reverse strand of the Minimal_CNTNN sequence used for EMSA analyses
NCTNN_FAM_fwd	[FAM]-CGTACGCAGACGAGACTCGTCTGCGCTACG	Forward strand of the Minimal_NCTNN sequence used for EMSA analyses (5' FAM label)
NCTNN_rev	CGTAGCGCAGACGAGTCTCGTCTGCGTACG	Reverse strand of the Minimal_NCTNN sequence used for EMSA analyses
Cons_long_fwd	CGTACTTAGGTTAGGCTAACCTAAGTCACG	Forward strand of the Consensus_Full sequence used for co-crystallization with $IdeR^{WT}$ and $IdeR^{P39G}$
Cons_long_rev	CGTGACTTAGGTTAGCCTAACCTAAGTACG	Reverse strand of the Consensus_Full sequence used for co-crystallization with $IdeR^{WT}$ and $IdeR^{P39G}$
Cons_short_fwd	CGTACTTAGGTTAGGCTAACCTAAGTACG	Forward strand of the Consensus_Full sequence used for co-crystallization with $IdeR^{Q43A}$
Cons_short_rev	CGTACTTAGGTTAGCCTAACCTAAGTACG	Reverse strand of the Consensus_Full sequence used for co-crystallization with $IdeR^{Q43A}$
C10S1_xtal_fwd	CGTACTTTGGTAAAGCTAACCTAAGTCACC	Forward strand of the cluster 10 site 1 sequence used for co-crystallization with $IdeR^{WT}$
C10S1_xtal_rev	GGTGACTTAGGTTAGCTTTACCAAAGTACG	Reverse strand of the cluster 10 site 1 sequence used for co-crystallization with $IdeR^{WT}$

Table S3. Root-mean square distance (RMSD) between aligned Cα atoms of different IdeR structures. Structures were superimposed using the Coot (5) secondary structure matching (SSM) algorithm. RMSD values are given in Å and the number of aligned residues is given in parentheses. $IdeR^{WT}$, $IdeR^{Q43A}$ and $IdeR^{P39G}$ refer to IdeR from *Saccharopolyspora erythraea* and engineered variants thereof (structures determined in this work). consDNA, consensus DNA; C10S1, cluster 10 site 1 DNA; *Mt*IdeR*, Mycobacterium tuberculosis* IdeR in complex with Co^{2+} and DNA (PDB ID 1U8R) (6); *Cd*DtxR, *Corynebacterium diphtheriae* DtxR in complex with Co^{2+} and DNA (PDB ID 1C0W) (7).

	**Co^{2+}-$IdeR^{WT}$**	**Co^{2+}-$IdeR^{WT}$ + consDNA**	**Fe^{2+}-$IdeR^{WT}$ + consDNA**	**Co^{2+}-$IdeR^{WT}$ + C10S1**	**Co^{2+}-$IdeR^{P39G}$ + consDNA**	**Co^{2+}-$IdeR^{Q43A}$ + consDNA**	**Co^{2+}-*Mt*IdeR + DNA**	**Co^{2+}-*Cd*DtxR + DNA**
**Co^{2+}-$IdeR^{WT}$**	-	0.76 (432)	0.71 (434)	0.79 (431)	0.77 (431)	0.80 (434)	1.30 (422)	1.53 (372)
**Co^{2+}-$IdeR^{WT}$ + consDNA**	0.76 (432)	-	0.30 (907)	0.34 (902)	0.58 (887)	0.41 (905)	1.20 (851)	1.26 (715)
**Fe^{2+}-$IdeR^{WT}$ + consDNA**	0.71 (434)	0.30 (907)	-	0.32 (902)	0.52 (889)	0.30 (912)	1.16 (859)	1.24 (712)
**Co^{2+}-$IdeR^{WT}$ + C10S1**	0.79 (431)	0.34 (902)	0.32 (902)	-	0.57 (882)	0.31 (902)	1.21 (853)	1.29 (717)
**Co^{2+}-$IdeR^{P39G}$ + consDNA**	0.77 (431)	0.58 (887)	0.52 (889)	0.57 (882)	-	0.67 (887)	1.16 (851)	1.40 (711)
**Co^{2+}-$IdeR^{Q43A}$ + consDNA**	0.80 (434)	0.41 (905)	0.30 (912)	0.31 (902)	0.67 (887)	-	1.22 (858)	1.24 (714)
**Co^{2+}-*Mt*IdeR + DNA**	1.30 (422)	1.20 (851)	1.16 (859)	1.21 (853)	1.16 (851)	1.22 (858)	-	1.35 (720)
**Co^{2+}-*Cd*DtxR + DNA**	1.53 (372)	1.26 (715)	1.24 (712)	1.29 (717)	1.40 (711)	1.24 (714)	1.35 (720)	-

Supplementary References

1. Zhou,T., Yang,L., Lu,Y., Dror,I., Dantas Machado,A.C., Ghane,T., Di Felice,R. and Rohs,R. (2013) DNAshape: a method for the high-throughput prediction of DNA structural features on a genomic scale. *Nucleic Acids Res.*, **41**, W56–W62.
2. Chiu,T.P., Rao,S., Mann,R.S., Honig,B. and Rohs,R. (2017) Genome-wide prediction of minor-groove electrostatic potential enables biophysical modeling of protein-DNA binding. *Nucleic Acids Res.*, **45**, 12565–12576.
3. Lavery,R., Moakher,M., Maddocks,J.H., Petkeviciute,D. and Zakrzewska,K. (2009) Conformational analysis of nucleic acids revisited: Curves+. *Nucleic Acids Res.*, **37**, 5917–5929.
4. Blanchet,C., Pasi,M., Zakrzewska,K. and Lavery,R. (2011) CURVES+ web server for analyzing and visualizing the helical, backbone and groove parameters of nucleic acid structures. *Nucleic Acids Res.*, **39**, W68–W73.
5. Emsley,P., Lohkamp,B., Scott,W.G. and Cowtan,K. (2010) Features and development of Coot. *Acta Crystallogr. Sect. D Biol. Crystallogr.*, **66**, 486–501.
6. Wisedchaisri,G., Holmes,R.K. and Hol,W.G.J. (2004) Crystal structure of an IdeR-DNA complex reveals a conformational change in activated IdeR for base-specific interactions. *J. Mol. Biol.*, **342**, 1155–1169.
7. Pohl,E., Holmes,R.K. and Hol,W.G.J. (1999) Crystal structure of a cobalt-activated diphtheria toxin repressor-DNA complex reveals a metal-binding SH3-like domain. *J. Mol. Biol.*, **292**, 653–667.
